# Development and Test-Item Analysis of a Freely Available 1900-Item Question Bank for Rheumatology Trainees

**DOI:** 10.7759/cureus.18382

**Published:** 2021-09-29

**Authors:** Bharat Kumar, Manish Suneja, Melissa L Swee

**Affiliations:** 1 Rheumatology, University of Iowa Hospitals and Clinics, Iowa City, USA; 2 Internal Medicine, University of Iowa Hospitals and Clinics, Iowa City, USA; 3 Nephrology, University of Iowa Hospitals and Clinics, Iowa City, USA

**Keywords:** test item analysis, graduate medical education, rheumatology, evaluation, medical education

## Abstract

Background

Tests composed of multiple-choice questions are an established tool to help evaluate knowledge of medical content. Within the field of rheumatology, there is an absence of free and easily-accessible sets of multiple-choice questions that have been rigorously evaluated and analyzed.

Objective

To develop a question bank composed of multiple-choice questions that evaluate trainee knowledge of rheumatology, as well as to investigate the psychometric properties (reliability, discrimination indices, difficulty indices) of items within the question bank.

Methods

Multiple-choice questions were drafted according to a strict methodology devised by the investigators. Between January and December 2020, questions were administered in sets of 20-25 questions to test-takers who were either current trainees or had recently graduated from training programs. Performance was evaluated through descriptive statistics (mean, median, range, standard deviation) and test-item statistics (difficulty index, discrimination index, reliability).

Results

Investigators drafted 1900 multiple choice questions within 45 sections each composed of 20 to 25 questions each. These questions were administered to 32 participants. The mean discrimination index was 0.57 (standard deviation: 0.22) and mean difficulty index was 0.38 (standard deviation: 0.23). Reliability indices for the 45 sections ranged from 0.45 to 0.85 (mean: 0.613, standard deviation: 0.09). The overall reliability index for the entire item bank was greater than 0.95.

Conclusion

The investigators developed a 1900-item question bank composed of items that have sufficient difficulty and discrimination indices to be used for low- and moderate-stakes settings. A rigorous methodology was employed to create the first freely-accessible reliable tool for the assessment of rheumatology knowledge. This tool can be purposed for both summative and formative evaluation in multiple settings and platforms.

## Introduction

Multiple-choice questions are a mainstay of educational assessment for the past century and are the basis for certification examinations in both Internal Medicine and Rheumatology, among other fields [1]. Tests composed of carefully-constructed multiple-choice questions have psychometric properties that make them conducive to learner assessment [2]. Additionally, when crafted appropriately, multiple-choice questions may enable self-regulated learning at the individual level [3].

Despite this, there is a lack of high-quality multiple-choice questions freely available for rheumatology fellows and other learners within the field. Currently available proprietary test item banks include the CARE (Continuing Assessment Review Evaluation) Modules)® (American College of Rheumatology, Atlanta, GA) the Medical Knowledge Self-Assessment Program® (American College of Physicians, Philadelphia, PA), UWorld® (UWorld, Dallas, TX), and NEJM Knowledge Plus (Massachusetts Medical Society, Waltham, MA). 

To address this need, the investigators have systematically developed a test item bank of 1900 items for Rheumatology fellows and other learners. Through the application of principles for test item writing, the investigators have ensured that these multiple-choice questions have psychometric properties conducive to their use for formative evaluation and self-evaluation.

## Materials and methods

The University of Iowa Institutional Review Board reviewed this project and determined it was not human subjects research since it was an educational intervention that only involved interactions involving an educational test and any disclosure of responses would not reasonably place the subjects at risk of criminal or civil liability or be damaging to the subjects’ financial standing, employability, educational advancement, or reputation. The project was completed from June 2017 to December 2020.

**Figure 1:**
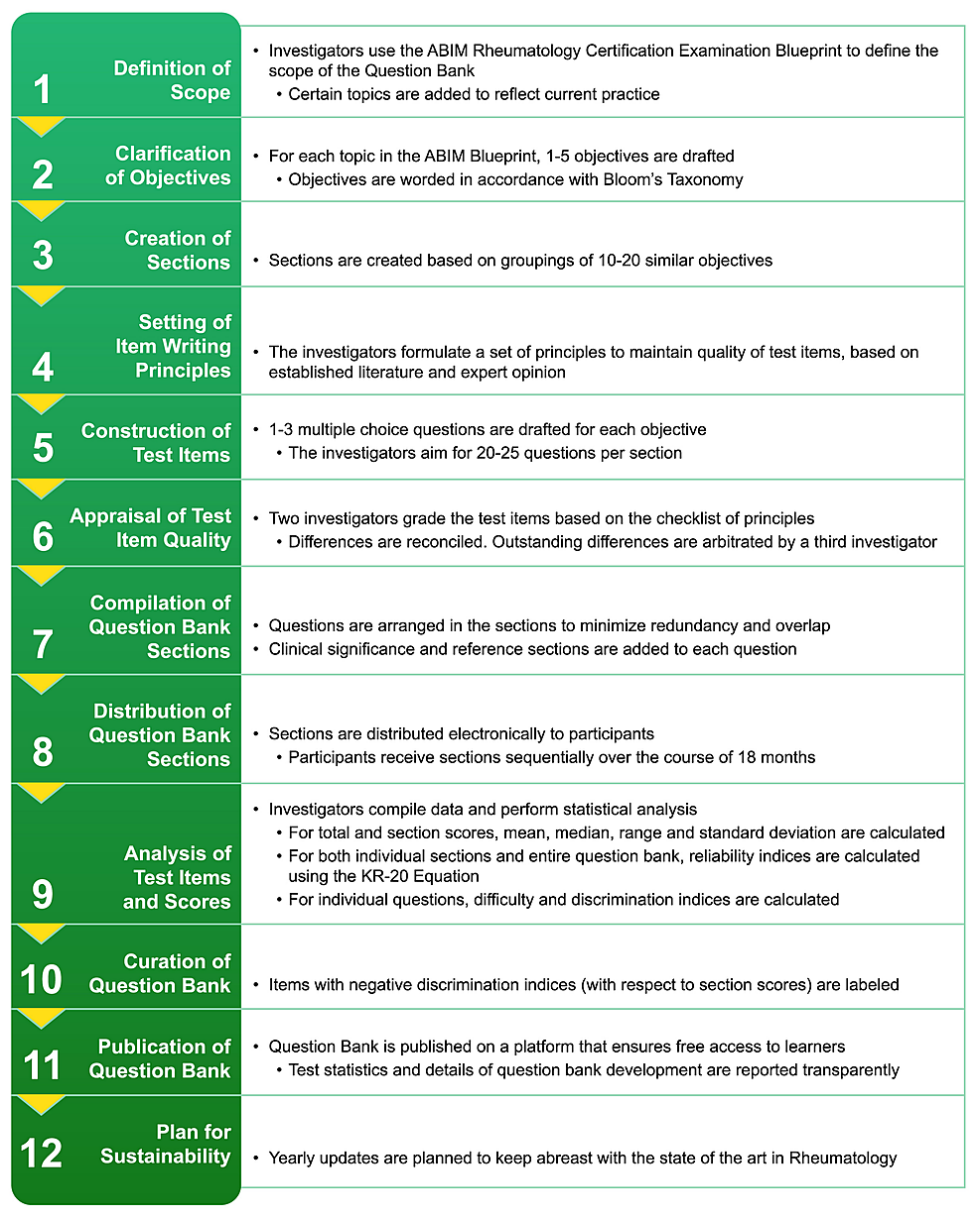
Stepwise process of creating and evaluating the test item bank.

A charter was drafted by the investigators who outlined the process of constructing and evaluating items (Figure 1). As part of the charter, the investigators delineated (1) the content of the item bank, (2) specific educational objectives, (3) appraisal of test item quality, (4) protocol for drafting test items, and (5) calculation of psychometric properties of test items. 

Content of the item bank

The scope of the item bank was defined by the American Board of Internal Medicine’s Blueprint for Rheumatology Certification [4]. Additionally, the authors incorporated three topics that have since become more important in clinical practice, including checkpoint immune therapy complications, cardiovascular complications of disease, and global epidemiology of rheumatology. Furthermore, approximately 70% of items were planned to be clinical in nature, with the remaining 30% being basic science, based on the investigators' desires to emphasize basic science as correlates of clinical practice.

Drafting of educational objectives

Educational objectives were drawn for each topic. Phrasing of the objectives was guided by Bloom’s Taxonomy, and objectives were classified into six categories: knowledge, comprehension, application, analysis, synthesis, and evaluation [5]. Two investigators independently assessed the specificity of objectives as well as the relevance to practice. In case of disagreement, a third investigator arbitrated the wording.

Based on the educational objectives, the number of sections was selected. As many as 20 related educational objectives were grouped together as part of a section, and up to three multiple-choice items were assigned based on a single objective.

Appraisal of test item quality

The investigators also developed a method for assessing the quality of test items. Using the National Board of Medical Examiner (NBME)’s Item Writing Manual, the Royal College of Physicians and Surgeons of Canada’s documentation on developing multiple-choice questions, a set of 10 criteria was drafted for each test item [6,7]. These included simplicity, objectivity, positivity, clarity, parallelism, brevity, relevance, order, independence, and appropriateness (Table 1). 

**Table 1:** Principles of drafting items. The authors outlined the process of constructing test items using 10 principles.

Criterion	Definition
Simplicity	The stem for each test item is first-order, i.e. minimizes the need to know other information apart from what is enquired
Objectivity	The test item has one unique correct answer (no none of the above, or all of the above answer choices)
Positivity	The stem for each test item enquires for the presence of an answer (no questions that test “exceptions”)
Clarity	The test item is tied to no more than two objectives
Parallelism	Each option is logically in parallel to one another
Brevity	Each stem and option has fewer than 210 characters, aside from technical vocabulary
Relevance	Each option represents a plausible option (no fictional answer choices)
Order	Each option is listed alphabetically, numerically, or otherwise logically
Independence	Each test item stands independently (no test items that share stems)
Appropriateness	The item is geared towards an advanced second-year rheumatology fellow

Item drafting

After the charter was ratified, the investigators began writing the components of each item: the stem, options (including correct answer choices and distractors), clinical significance, and references.

The abovementioned set of 10 criteria guided the production of these test items. For each item, only one correct answer was listed, along with two other, incorrect answer choices (distractors). A fourth answer choice, “I will have to look that up,” was also added. This form of test item format was selected due to previous data demonstrating equivalent psychometric properties of three-choices compared to four-choices [8]. Additionally, the fourth answer choice was incorporated to minimize random guessing and promote reflection on confidence of knowledge [9].

Each test item also had an explanation, entitled “Clinical Significance” that justified the importance of knowing this particular piece of information in the clinical context. When feasible, explanations also included discussions about the relationships between the distractors, the correct answer choice, and the stem.

In addition, each explanation included a citation to a peer-reviewed source or to information published directly by a regulatory agency. Inclusion of references to freely accessible narrative and systematic review articles were prioritized due to their greater accessibility and broader scope.

After completing each section, the investigators reconvened and evaluated test items to determine how they satisfied the 10 criteria. Two separate investigators graded the test items. Any items below a score of 8 from either of the two investigators were either reworded or eliminated entirely. Kappa scores were utilized to assess the inter-rater reliability with respect to each of the 10 criteria.

Participant selection

Thirty-two participants were selected via convenience sampling. These individuals were known to the investigators as either current rheumatology fellows, recent graduates, or advanced Internal Medicine resident physicians. Participants had to have regular access to the internet and a valid electronic mail address. To promote adherence, participants were told that answers and explanations would only be provided once the investigators had received the participant answer selections.

Examination

Once completed, the 1900 test items were collated into a series of word documents and distributed individually via electronic mail to 32 participants. Participants were encouraged to complete each section within a week’s time and return answers to obtain the next section. They were also encouraged to use the ‘honor code’ and answer without access to other resources. Lastly, they were also asked to avoid guessing and either use the ‘D’ answer choice ("I will have look that up") or skip the question altogether whenever they were unsure of the correct answer. The 'D' answer choice is intended to help flag items that test-takers would like to look up later rather than guess. Once a section was completed by all participants, explanations, objectives, and references were provided to the participant.

Data analysis

Responses were collected analyzed using Microsoft Excel® (Redmond, Washington). Four levels of data analysis were performed.

For the entire item bank, the mean, median, mode and standard deviation (SD) for scores were calculated. The investigators also calculated the Cronbach-alpha (using the Kuder-Richardson 20 Equation) for reliability. Likewise, for each test section, the mean, median, mode, standard deviation, and reliability indices (Cronbach alpha) were tabulated. Additionally, the investigators calculated the mean and standard deviation for each of the 95 reliability indices.

At the individual item level, the difficulty and discrimination indices were determined, relative to (1) the total score, and (2) the section score. The mean and standard deviations of these difficulty and discrimination indices were subsequently calculated.

Lastly, the difficulty and discrimination indices were determined relative to the taxon in which the objective was grouped. When calculating the test statistics, the answer choice ‘D’ was counted as being answered incorrectly.

## Results

Participant characteristics

There were 32 participants that completed all 1900 questions. Among the 32, three were Internal Medicine resident physicians, 22 were current rheumatology fellows, six had graduated rheumatology fellowship within the previous year, and one were board-certified rheumatologists who had graduated rheumatology fellowship more than one year prior to the project. These participants were from seven different institutions in five different states.

Test item content

A bank of 1900 multiple-choice test items were drafted by the investigators, which were divided into 95 sections composed of 20-25 questions each. The item banks are freely available online in two forms: (1) The Learner's Guide, which only has the multiple-choice questions, and (2) the Complete Guide, which has multiple-choice questions, explanations, and references [10,11].

Seven sections, accounting for 143 questions (7.52%), focused exclusively on an aspect of basic science, such as immunology, laboratory techniques, anatomy, or pharmacodynamics, in line with the ABIM Blueprint for the Rheumatology Certification Examination (7%). Another 367 questions had objectives that significantly incorporated basic science into the clinical setting (19.30%). Altogether, these constituted 510 questions, accounting for 26.83%, in line with our plans to have approximately 30% of questions addressing basic science.

These questions were based on 1238 distinct objectives. Objectives were split among the six levels of Bloom Taxonomy. A plurality of test items involved comprehension, followed by application and analysis of knowledge (40.03%, 23.51% and 15.89%, respectively). Slightly over 5% of questions involved synthesis (5.16%).

Appraisal of test item quality

All 1900 questions underwent two rounds of review by three investigators. The average score for test items during the initial review was 9.1 (SD=0.8). The kappa-statistic was 0.94. 150 questions were below the threshold of 8 and so were re-written in the second round of reviews. The second round of reviews yielded an average score of 9.4 (SD=0.3). The kappa-statistic was 0.98. The greatest source of disagreement was parallelism among answer choices, which was the source of disagreement in 132 of the 1900 questions (6.9%). Table 2 displays the inter-rater reliabilities for each criterion upon final review.

**Table 2:** Inter-rater reliability for each criterion upon final review of the 1900 test Items.

Criterion	Inter-rater reliability (K) at final review
Simplicity	0.96
Objectivity	0.99
Positivity	0.94
Clarity	0.99
Parallelism	0.98
Brevity	1.00
Relevance	0.99
Order	1.00
Independence	1.00
Appropriateness	0.95

Test item statistics

Test item statistics were calculated for each of the 95 sections as well as the entire test item bank (Table 3). For the entire item bank, the mean score was 0.58 (Range: 0.31-0.69; median: 0.608; SD=0.083). Reliability of the entire question bank, calculated through the KR-20, was 0.986.

**Table 3:** Mean scores, median values, and reliability of test item bank sections.

Topic	Number of Questions	Mean Score	Median Score	Minimum	Maximum	Standard Deviation	Reliability
Principles of Clinical Examination	20	0.708	0.750	0.3	0.9	0.149	0.641
Hand and Wrist Examination	23	0.661	0.667	0.33	0.833	0.125	0.522
Elbow and Shoulder Examination	23	0.64	0.652	0.261	0.826	0.131	0.518
Spine Examination	22	0.133	0.727	0.318	0.909	0.133	0.520
Hip and SI Joint Examination	20	0.717	0.750	0.4	0.95	0.146	0.543
Knee Examination	20	0.664	0.700	0.5	0.85	0.147	0.612
Foot & Ankle Examination	20	0.589	0.625	0.1	0.9	0.171	0.659
Eye Examination	21	0.713	0.762	0.143	0.905	0.166	0.725
Nerve Entrapment Syndromes	20	0.697	0.700	0.15	0.95	0.18	0.759
Peripheral Nervous System Disorders	20	0.813	0.850	0.4	1	0.157	0.762
Fibromyalgia & Central Pain Sensitivity Syndromes	20	0.734	0.750	0.15	1	0.176	0.768
Autoimmune Eye Disease	20	0.747	0.800	0.2	0.95	0.154	0.703
Autoimmune Ear Disease	20	0.669	0.750	0.1	1	0.214	0.834
Panniculitis and Dermatitis	20	0.7	0.775	0.05	0.95	0.218	0.850
Peripheral Vascular Diseases	22	0.805	0.864	0.182	1	0.183	0.845
Rheumatologic Manifestations of Kidney Disease	20	0.644	0.700	0.35	0.9	0.154	0.600
Rheumatologic Manifestations of Endocrine Disease	20	0.722	0.750	0.2	0.9	0.162	0.720
Rheumatologic Manifestations of GI Disease (except IBD)	20	0.767	0.800	0.25	0.095	0.173	0.772
Rheumatologic Manifestations of CNS Disease	20	0.652	0.675	0.15	0.9	0.183	0.715
Principles of Laboratory Examination	20	0.756	0.775	0.35	0.95	0.137	0.635
Principles of Radiographic Examination	20	0.773	0.800	0.4	1	0.14	0.639
Innate Immune System	20	0.67	0.700	0.4	0.9	0.132	0.495
Adaptive Immune System	20	0.675	0.750	0.1	0.95	0.228	0.845
Immunodeficiencies	20	0.669	0.725	0.15	0.9	0.196	0.780
Sarcoidosis and Miscellaneous Rheumatologic Conditions	20	0.722	0.750	0.3	0.9	0.132	0.560
Steroids	20	0.717	0.750	0.3	0.95	0.167	0.730
NSAIDs and Colchicine	20	0.761	0.800	0.3	0.9	0.149	0.675
Principles of Rheumatologic Disease Management	20	0.728	0.750	0.35	0.95	0.14	0.586
Methotrexate and Leflunomide	20	0.78	0.800	0.3	0.95	0.139	0.649
Sulfasalazine and Hydroxychloroquine	25	0.706	0.580	0.2	0.76	0.185	0.811
Azathioprine and Mycophenolate Mofetil	20	0.686	0.750	0.15	0.9	0.187	0.759
Cyclophosphamide, Cyclosporine, and Tacrolimus	20	0.697	0.700	0.2	0.95	0.167	0.720
Urate-Lowering Therapy	21	0.796	0.810	0.333	1	0.15	0.726
Other Conventional DMARDs	20	0.645	0.700	0.25	0.95	0.174	0.703
TNF Inhibitors	20	0.748	0.800	0.25	0.95	0.162	0.723
Drugs Targeting IL1, IL12, IL17 and IL23	20	0.699	0.725	0.25	0.95	0.163	0.682
Rituximab, Belimumab, and Tocilizumab	20	0.761	0.775	0.35	0.95	0.127	0.587
Abatacept, Apremilast, Mepolizumab, and Jakinibs	20	0.692	0.750	0.2	0.9	0.16	0.675
Metabolic and Crystalline Diseases	20	0.518	0.500	0.091	1	0.192	0.710
Gout	20	0.659	0.650	0.2	0.95	0.169	0.714
Calcium Pyrophosphate Arthritis	21	0.597	0.625	0.25	0.9	0.162	0.634
Osteoarthritis	20	0.55	0.550	0.2	0.85	0.154	0.621
Osteoporosis	23	0.656	0.667	0.291	0.875	0.158	0.650
Paget Disease, Osteonecrosis, Osteomalacia, and HCTDs	24	0.572	0.600	0.16	0.8	0.137	0.454
Ankylosing Spondylitis	20	0.73	0.700	0.35	1	0.15	0.632
Reactive Arthritis	20	0.614	0.650	0.15	0.95	0.166	0.670
Psoriatic Arthritis	20	0.591	0.625	0.15	0.85	0.154	0.600
IBD-associated Arthritis	20	0.575	0.600	0.15	0.9	0.163	0.610
RA: Pathogenesis, Prognosis, and Epidemiology	20	0.581	0.600	0.2	0.8	0.152	0.565
RA: Diagnosis and Management Strategies	20	0.583	0.600	0.3	0.85	0.15	0.547
RA: Extra-articular Manifestations	20	0.594	0.625	0.25	0.8	0.158	0.617
SLE: Pathogenesis, Diagnosis, and Epidemiology	20	0.57	0.550	0.25	0.8	0.161	0.635
Cutaneous Manifestations of Lupus	21	0.555	0.571	0.19	0.86	0.163	0.627
Lupus Nephritis	20	0.569	0.600	0.25	0.85	0.164	0.613
APLS and SLE in Reproductive Health	20	0.539	0.600	0.15	0.75	0.645	0.531
Systemic Sclerosis: Pathogenesis, Diagnosis, and Epidemiology	22	0.486	0.500	0.136	0.727	0.147	0.592
Visceral Manifestations of Systemic Sclerosis	20	0.402	0.400	0.05	0.65	0.167	0.648
Localized Scleroderma and Ranaud Phenomenon	20	0.35	0.350	0.1	0.65	0.152	0.602
Scleroderma Mimics	24	0.396	0.417	0.083	0.708	0.139	0.570
Inflammatory Myositis: Prognosis and Treatment	21	0.402	0.381	0.095	0.667	0.154	0.590
Inflammatory Myositis: Pathophysiology and Diagnosis	23	0.463	0.478	0.217	0.696	0.135	0.507
Myositis Mimics	20	0.42	0.400	0.15	0.7	0.15	0.530
Sjogren Syndrome	20	0.511	0.500	0.2	0.8	0.159	0.620
MCTD, UCTD, and Overlap Syndromes	20	0.519	0.500	0.15	0.85	0.146	0.496
Giant Cell Arteritis	20	0.559	0.600	0.25	0.8	0.146	0.532
PMR & Takayasu Arteritis	23	0.467	0.457	0.13	0.794	0.141	0.541
Polyarteritis Nodosa	20	0.44	0.450	0.1	0.7	0.147	0.505
Variable Vessel Vasculitides and Thromboangiitis Obliterans	20	0.422	0.400	0.2	0.8	0.155	0.553
Vasculitis Mimics	20	0.534	0.550	0.3	0.85	0.138	0.520
Primary CNS Vasculitis	20	0.508	0.500	0.2	0.75	0.146	0.511
ANCA-Associated Vasculitis: Diagnosis & Clinical Presentation	20	0.439	0.425	0.15	0.75	0.146	0.509
ANCA-Associated Vasculitis: Associated Diseases, Prognosis & Treatment	20	0.363	0.350	0.1	0.7	0.162	0.650
Cutaneous Small Vessel Vasculitides (except IgA Vasculitis)	20	0.405	0.375	0.15	0.8	0.17	0.661
IgA Vasculitis	20	0.413	0.400	0.1	0.8	0.152	0.586
Cryoglobulinemia and Related Diseases	20	0.431	0.400	0.2	0.75	0.162	0.658
Relapsing Polychondritis	20	0.405	0.350	0.1	0.8	0.176	0.670
IgG4-Related Disease	21	0.551	0.524	0.238	0.857	0.146	0.535
Juvenile Idiopathic Arthritis	20	0.494	0.450	0.2	0.9	0.176	0.677
Kawasaki Disease	20	0.475	0.450	0.15	0.8	0.176	0.699
Periodic Fever Syndromes	20	0.381	0.350	0.15	0.8	0.159	0.597
Autoinflammatory Syndromes	20	0.383	0.375	0.15	0.65	0.154	0.685
Septic Arthritis & Osteomyelitis	20	0.444	0.450	0.2	0.8	0.138	0.514
Lyme Disease	20	0.475	0.450	0.2	0.8	0.161	0.643
Whipple Disease	20	0.327	0.300	0.05	0.6	0.158	0.613
Rheumatic Fever & Post-Infectious Reactive Arthritis	20	0.395	0.400	0.15	0.75	0.155	0.588
Disseminated Gonococcemia	20	0.384	0.400	0.1	0.7	0.15	0.561
Mycobacterial Infections	20	0.569	0.600	0.25	0.85	0.141	0.517
Fungal Infections	20	0.384	0.350	0.05	0.7	0.153	0.563
Human Immunodeficiency Virus Infection	20	0.336	0.300	0.05	0.65	0.136	0.538
Viral Arthritides	20	0.366	0.350	0.1	0.75	0.17	0.650
Benign Lymphoproliferative & Hematologic Disorders	20	0.341	0.350	0.05	0.75	0.157	0.598
Paraneoplastic & Chemotherapeutic Agents	20	0.377	0.350	0.15	0.8	0.17	0.650
Bony & Cartilaginous Neoplasms	20	0.431	0.450	0.15	0.7	0.164	0.593
Total	1900	0.576	0.608	0.05	1	0.083	0.986

Mean scores for each section varied from 0.133 (Spine Examination) to 0.813 (Peripheral Nervous System Disorders). Altogether, these centered around a median of 0.575 and a mean of 0.569 (SD=0.145). Reliability indices for sections ranged from 0.454 (Paget Disease) to 0.850 (Panniculitis and Dermatitis). The mean reliability index was 0.632, which was similar to the median of 0.627. The standard deviation of these reliability indices was 0.090.

**Figure 2:**
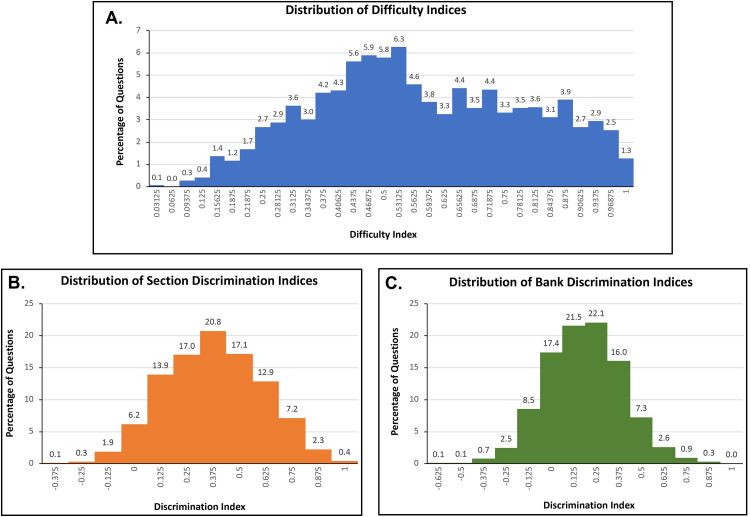
Distribution of difficulty and discrimination indices for each test item.

Difficulty and discrimination indices for each test item was also calculated relative to its contribution to the section score and to the total score (Table 4). Difficulty ranged from 0 to 1.00 (mean: 0.576; median: 0.563; SD: 0.217). Discrimination indices relative to section scores ranged from -0.375 to 1 (mean: 0.376; SD: 0.235), with a median of 0.375. In contrast, discrimination indices relative to the total score ranged from -0.625 to 0.875 (mean: 0.184; SD: 0.214) with a median of 0.375 (Figure 2).

**Table 4:** Difficulty indices and discrimination indices.

Topic	Question	Difficulty Index	Section Discrimination Index	Bank Discrimination Index
Principles of Clinical Examination	1	0.8125	0.5	0
	2	0.9375	0.25	-0.125
	3	0.53125	0.375	0
	4	0.4375	0.25	-0.25
	5	0.6875	0.375	0
	6	0.90625	0.375	0.125
	7	0.8125	0.375	0.125
	8	0.53125	0.25	0.25
	9	0.59375	0.5	-0.125
	10	0.875	0.25	0
	11	0.375	0.625	0.375
	12	0.875	0.5	0.125
	13	0.78125	0.25	0
	14	0.90625	0.375	0.125
	15	0.875	0.25	-0.25
	16	0.4375	0.375	0.125
	17	0.65625	0.375	0
	18	0.90625	0.25	0
	19	0.71875	0.5	0.25
	20	0.5	0.375	-0.25
Hand and Wrist Examination	1	0.875	0.375	0.125
	2	0.46875	0.375	-0.25
	3	0.53125	0.5	0.25
	4	0.40625	0.5	-0.125
	5	0.84375	0.25	0.375
	6	0.5	0.25	-0.125
	7	0.46875	0.375	0
	8	0.40625	0.375	0.375
	9	0.59375	0.75	0
	10	0.34375	0.375	0.125
	11	0.59375	0.25	0.125
	12	0.53125	0.25	0.125
	13	0.8125	0.375	0.125
	14	0.875	0.25	-0.25
	15	0.46875	0.5	0
	16	0.84375	0.25	-0.25
	17	0.78125	0.25	0.25
	18	0.875	0.25	0.125
	19	0.9375	0	0.25
	20	0.75	0.375	0
	21	0.53125	0.25	-0.25
	22	0.90625	0.375	0.25
	23	0.875	0.25	0.125
	24	0.65625	0	-0.125
Elbow and Shoulder Examination	1	0.78125	0.375	0.375
	2	0.375	0.125	0.125
	3	0.375	0.125	-0.25
	4	0.375	0.375	0.375
	5	0.4375	0.75	0.375
	6	0.59375	0.125	-0.125
	7	0.46875	0.5	0.375
	8	0.75	0.375	-0.125
	9	0.84375	0.25	0.125
	10	0.5625	0.625	0.125
	11	0.53125	0.625	0.5
	12	0.40625	0.25	0
	13	0.53125	0.375	0.25
	14	0.78125	0.25	-0.125
	15	0.78125	0.125	0
	16	0.4375	0.125	-0.125
	17	0.71875	-0.125	0.25
	18	0.875	0.25	0
	19	0.8125	0.25	0.125
	20	0.875	0.125	0.125
	21	0.8125	0.25	-0.125
	22	0.875	0.375	0.25
Spine Examination	1	0.53125	0.5	0.375
	2	0.5	0.75	0.375
	3	0.65625	0.375	-0.125
	4	0.75	0.375	0.375
	5	0.65625	0	0.125
	6	0.875	0.375	0.375
	7	0.8125	0.375	0.375
	8	0.71875	0.5	0.25
	9	0.5625	0.5	0.5
	10	0.84375	0.125	0.25
	11	0.8125	0.125	0.125
	12	0.5625	0.25	-0.125
	13	0.8125	0.375	0.5
	14	0.84375	0	0
	15	0.84375	0.25	0.125
	16	0.65625	0.375	0.25
	17	0.75	0.125	0
	18	0.5	0.5	0
	19	0.75	0.375	0.25
	20	0.875	0.375	0.125
	21	0.96875	0.125	0.125
	22	0.59375	0.25	0.125
Hip and SI Joint Examination	1	0.5625	0.625	0.375
	2	0.53125	0.625	0.25
	3	0.6875	0.125	0
	4	0.78125	0.25	0.25
	5	0.90625	0.125	0.25
	6	0.4375	0.375	0.375
	7	0.84375	0.125	0
	8	0.75	0.5	0.25
	9	0.53125	0.125	0.125
	10	0.875	0.25	0
	11	0.53125	0.5	0.5
	12	0.5	0.625	0.625
	13	0.9375	0.125	0.25
	14	0.96875	0.125	0
	15	0.8125	0.25	0.25
	16	0.53125	0.375	0.25
	17	0.875	0.5	0.125
	18	0.71875	0.75	0.125
	19	0.9375	0.125	0
	20	0.65625	0.625	0.25
Knee Examination	1	0.875	0.375	0.375
	2	0.84375	0.375	0.25
	3	0.71875	0.25	0.125
	4	0.46875	0.125	0.125
	5	0.875	0.5	0.5
	6	0.6875	0.5	0.125
	7	0.65625	0.625	0.25
	8	0.5625	0.375	0.625
	9	0.25	0.5	0.375
	10	0.40625	0.125	0
	11	0.46875	0.375	0.25
	12	0.375	0.375	0.25
	13	0.4375	0.625	0.25
	14	0.75	0.625	0.375
	15	0.875	0.25	-0.125
	16	0.6875	0.375	0
	17	0.84375	0.375	0.125
	18	0.59375	0	-0.25
	19	0.96875	0.125	0.125
	20	0.9375	0.25	0.25
Foot & Ankle Examination	1	0.84375	0.5	0.375
	2	0.6875	0.5	0.25
	3	0.75	0.375	0.125
	4	0.59375	0.125	0.125
	5	0.46875	0.75	0.375
	6	0.65625	0.625	0.25
	7	0.625	0.75	0.25
	8	0.4375	-0.125	0.625
	9	0.46875	0.125	-0.125
	10	0.5	0	0.25
	11	0.6875	0.5	0.375
	12	0.4375	0.875	0
	13	0.4375	0.625	0.125
	14	0.4375	0.5	0.25
	15	0.78125	0.125	0.125
	16	0.4375	0.375	0
	17	0.375	0.125	0.375
	18	0.53125	0.625	0
	19	0.78125	0.125	0.125
	20	0.84375	0.375	0.5
Eye Examination	1	0.875	0.375	0.375
	2	1	0	0
	3	0.875	0.375	0.375
	4	0.84375	0.5	0.25
	5	0.4375	0.375	0.5
	6	0.53125	0.75	0.125
	7	0.65625	0.5	0.375
	8	0.4375	0	-0.125
	9	0.8125	0.25	0.125
	10	0.53125	0.25	-0.125
	11	0.53125	0.25	0.25
	12	0.5	0.25	0
	13	0.84375	0.25	0
	14	0.78125	0.25	0.5
	15	0.78125	0.125	0.375
	16	0.78125	0.375	0.25
	17	0.65625	0.5	0.25
	18	0.71875	0.625	0.25
	19	0.78125	0.625	0.375
	20	0.9375	0.25	0.125
	21	0.65625	0.625	0.25
Nerve Entrapment Syndromes	1	0.96875	0.125	0.125
	2	0.8125	0.375	0.375
	3	0.78125	0.625	0.5
	4	0.5	0.25	-0.125
	5	0.5625	0.25	0.625
	6	0.4375	0.375	0
	7	0.8125	0.625	0.375
	8	0.78125	0.25	0.5
	9	0.875	0.375	0.25
	10	0.40625	0.75	0.125
	11	0.84375	0.375	0.25
	12	0.78125	0.75	0.375
	13	0.53125	0.25	0.125
	14	0.5	0.75	0.125
	15	0.90625	0.25	0
	16	0.75	0.25	0.25
	17	0.78125	0.25	0.25
	18	0.5	0.625	0.75
	19	0.53125	0.5	0.5
	20	0.875	0.25	0.25
Peripheral Nervous System Disorders	1	1	0	0
	2	0.8125	0.125	0.125
	3	0.6875	0.875	-0.125
	4	0.875	0	0
	5	0.90625	0	0
	6	0.59375	0.625	0.25
	7	0.59375	0.875	0
	8	1	0	0
	9	0.875	0.25	0.25
	10	0.9375	0.25	0.25
	11	0.90625	0.375	0.25
	12	0.90625	0.375	0.25
	13	0.84375	0.375	0
	14	0.5625	0.75	0.25
	15	0.96875	0.125	0
	16	0.5625	1	0.25
	17	0.6875	0.375	0
	18	0.96875	0.125	0.125
	19	0.875	0.375	0.375
	20	0.6875	0.5	0.5
Fibromyalgia & Central Pain Sensitivity Syndromes	1	0.90625	0.375	0.25
	2	0.53125	0.875	0
	3	0.75	0.625	0
	4	0.8125	0.5	0.375
	5	0.90625	0.375	0.25
	6	0.71875	0.25	0.125
	7	0.5	1	0.25
	8	0.5625	0.625	0.375
	9	0.5625	0.875	0.125
	10	0.9375	0.125	0.125
	11	0.5625	0.375	-0.25
	12	0.875	0	0.125
	13	0.9375	0.125	0.125
	14	0.8125	0.5	0.375
	15	0.59375	0.375	0.125
	16	0.5625	0.25	0.375
	17	0.9375	0.25	0.25
	18	0.9375	0.125	0.125
	19	0.78125	0.25	0.125
	20	0.5	0.375	-0.25
Autoimmune Eye Disease	1	1	0	0
	2	0.59375	0.625	0.5
	3	0.5625	0.75	0.75
	4	0.875	0.125	0.25
	5	0.875	0.375	0.125
	6	0.59375	0.5	0
	7	0.96875	0.125	0.125
	8	0.875	0.375	0.125
	9	0.8125	0.375	0.375
	10	0.96875	0.125	0.125
	11	0.90625	0.375	0.375
	12	0.90625	0.25	0.125
	13	0.46875	0.25	0.125
	14	0.53125	0.125	0
	15	0.71875	0.625	0.5
	16	0.5625	0.625	0.5
	17	0.96875	0.125	0.125
	18	0.53125	0.375	0.25
	19	0.6875	0.25	-0.125
	20	0.53125	0.75	0.375
Autoimmune Ear Disease	1	0.5625	0.375	0
	2	0.875	0.5	0.5
	3	0.46875	0.75	0.625
	4	0.71875	0.75	0.625
	5	0.84375	0.625	0.375
	6	0.75	0.5	0.25
	7	0.78125	0.375	0.25
	8	0.8125	0.625	0.5
	9	0.40625	0.5	0.25
	10	0.4375	0.75	0.25
	11	0.4375	0.75	0.25
	12	0.46875	0.625	0.625
	13	0.78125	0.625	0.375
	14	0.71875	0.375	0.375
	15	0.90625	0.25	0.125
	16	0.78125	0.5	0.375
	17	0.46875	0.5	0.125
	18	0.4375	0.5	0
	19	0.90625	0.125	-0.125
	20	0.8125	0.375	0.125
Panniculitis and Dermatitis	1	0.5625	0.875	0.5
	2	0.5625	0.625	0.625
	3	0.59375	0.625	0.375
	4	0.5625	0.625	0.375
	5	0.875	0.5	0.25
	6	0.5625	0.5	0.375
	7	0.5	0.625	0.5
	8	0.75	0.75	0.25
	9	0.46875	0.5	0.25
	10	0.53125	0.625	0.5
	11	0.875	0.5	0.375
	12	0.96875	0.125	0.125
	13	0.8125	0.375	0.125
	14	0.9375	0.25	0.25
	15	0.78125	0.25	0
	16	0.46875	0.5	0.25
	17	0.875	0.375	0.25
	18	0.6875	0.625	0.25
	19	0.71875	0.375	0.125
	20	0.90625	0.25	0.25
Peripheral Vascular Diseases	1	0.46875	0.25	0
	2	0.71875	0.375	0.25
	3	0.9375	0.25	0.125
	4	0.71875	0.5	0.375
	5	0.65625	0.875	0.125
	6	0.75	0.625	0.5
	7	0.875	0.375	0.25
	8	0.84375	0.125	0.375
	9	0.90625	0.375	0.375
	10	0.9375	0.25	0.25
	11	1	0	0
	12	0.84375	0.375	0
	13	0.75	0.375	0.375
	14	0.875	0.25	0
	15	0.78125	0.5	0.25
	16	0.65625	0.375	0.25
	17	0.8125	0.375	0.25
	18	0.84375	0.5	0.375
	19	0.875	0.125	0.125
	20	0.9375	0.25	0.25
	21	0.71875	0.625	0.125
	22	0.8125	0.5	0.125
Rheumatologic Manifestations of Kidney Disease	1	0.46875	0.875	0
	2	0.71875	0.125	0
	3	0.875	0.375	0
	4	0.84375	0.375	-0.125
	5	0.8125	0.625	0
	6	0.5	0	0
	7	0.5625	0.625	0.25
	8	0.84375	0.125	-0.125
	9	0.65625	0.25	-0.25
	10	0.78125	0.625	-0.125
	11	0.71875	0.125	-0.125
	12	0.46875	0.5	0
	13	0.5	0.25	-0.125
	14	0.75	0.375	0
	15	0.46875	0.375	-0.125
	16	0.59375	0.75	-0.125
	17	0.40625	0.75	0.375
	18	0.4375	0	0.125
	19	0.625	0.125	-0.125
	20	0.84375	0.625	-0.375
Rheumatologic Manifestations of Endocrine Disease	1	0.5625	0.125	0.375
	2	0.65625	0.375	0.25
	3	0.90625	0.125	0.125
	4	0.40625	0.375	0.125
	5	0.9375	0.25	0.25
	6	0.53125	0.625	0.625
	7	0.9375	0.125	0.125
	8	0.9375	0.25	0.25
	9	0.6875	0.25	0.125
	10	0.78125	0.5	0.5
	11	0.90625	0.25	0.125
	12	0.46875	0.75	0.375
	13	0.71875	0.5	0.5
	14	0.90625	0.375	0.25
	15	0.53125	0.375	0.25
	16	0.9375	0.125	0.125
	17	0.65625	0.75	0.5
	18	0.5	0.5	0.625
	19	0.53125	0.375	0.25
	20	0.9375	0.25	0.25
Rheumatologic Manifestations of GI Disease (except IBD)	1	0.75	0.5	0.125
	2	0.9375	0.25	0.25
	3	0.53125	0.25	0.125
	4	0.65625	0.5	0.875
	5	0.875	0.375	0.25
	6	0.90625	0.25	0.25
	7	0.53125	0.5	0
	8	0.65625	0.625	0.5
	9	0.65625	0.75	0.125
	10	0.75	0.625	0.25
	11	0.875	0.25	0.125
	12	0.625	0.875	0.375
	13	0.90625	0.125	0.125
	14	0.8125	0.625	0.5
	15	0.65625	0.375	0.25
	16	0.9375	0	0.125
	17	0.90625	0.125	0.25
	18	0.84375	0.375	0.25
	19	0.625	0.25	0.125
	20	0.90625	0.25	0.25
Rheumatologic Manifestations of CNS Disease	1	0.8125	0.25	0.375
	2	0.59375	-0.125	0.375
	3	0.78125	0.5	0.25
	4	0.40625	0.25	0.125
	5	0.6875	0.125	0.25
	6	0.625	0.5	0.125
	7	0.53125	0.375	0.375
	8	0.75	0.625	0.25
	9	0.59375	0.375	0.25
	10	0.5625	0.5	0.125
	11	0.53125	0.5	0.25
	12	0.65625	0.375	0.375
	13	0.71875	0.875	0.25
	14	0.65625	0.75	0.25
	15	0.75	0.5	0
	16	0.71875	0.125	0.25
	17	0.65625	0.875	0.5
	18	0.8125	0.375	0.125
	19	0.6875	0.5	0.5
	20	0.5	0.625	0.375
Principles of Laboratory Examination	1	0.4375	0.25	0.125
	2	0.65625	0.5	0.25
	3	0.71875	0.375	-0.125
	4	0.8125	0.375	0.125
	5	0.84375	0.375	0.25
	6	0.875	0.25	0
	7	0.4375	0.625	0.25
	8	0.8125	0.125	0.125
	9	1	0	0
	10	0.90625	0.125	0.125
	11	0.5	0.25	-0.25
	12	0.96875	0.125	0.125
	13	0.5625	0.875	0
	14	0.75	0.5	0
	15	0.71875	0.625	0.125
	16	0.9375	0.25	0.125
	17	0.90625	0.25	0.25
	18	0.96875	0.125	0.125
	19	0.4375	0.75	0
	20	0.875	0.125	0
Principles of Radiographic Examination	1	0.6875	0.75	0.375
	2	0.9375	0.25	0
	3	0.78125	0.25	0
	4	0.5625	0.875	0.375
	5	0.875	0.375	0.25
	6	0.9375	0.25	0.125
	7	0.90625	0.25	0.25
	8	0.9375	0	0
	9	0.84375	0.5	0.375
	10	0.71875	0.375	0.125
	11	0.96875	0.125	0.125
	12	0.78125	0.25	0.375
	13	0.53125	0.75	0.875
	14	0.875	0.125	0.25
	15	0.5625	0.5	0.125
	16	0.84375	0.25	0.25
	17	0.53125	0.5	0.125
	18	0.84375	0.125	0.25
	19	0.65625	0.5	0.125
	20	0.6875	0	-0.125
Innate Immune System	1	0.53125	0.875	-0.125
	2	0.5	0.625	-0.125
	3	0.53125	0.75	0.25
	4	0.84375	-0.125	0
	5	0.5	0.375	0.375
	6	0.84375	0.125	0.125
	7	0.59375	-0.125	0.375
	8	0.5	0.375	-0.125
	9	0.5	0.25	0.25
	10	0.65625	0.75	-0.125
	11	0.8125	0.5	0.125
	12	0.71875	0.25	-0.125
	13	0.9375	0.125	0.25
	14	0.5625	0.375	0.625
	15	0.5625	0.25	-0.125
	16	0.4375	0.125	0.25
	17	0.78125	0.375	0.25
	18	0.90625	0.125	0
	19	0.84375	0.5	0.25
	20	0.84375	0	0
Adaptive Immune System	1	1	0	0
	2	0.5625	0.625	0.125
	3	0.71875	0.25	0
	4	0.5	0.875	0.5
	5	0.75	0.125	-0.25
	6	0.71875	0.625	0.25
	7	0.75	0.625	0.25
	8	0.78125	0.5	0.375
	9	0.65625	0.875	0.625
	10	0.53125	0.875	0.25
	11	0.65625	0.25	0
	12	0.8125	0.5	0.125
	13	0.6875	0.25	0.25
	14	0.53125	0.75	0.625
	15	0.53125	0.75	0.25
	16	0.6875	0.625	0.375
	17	0.59375	0.5	0.375
	18	0.5625	0.875	0.25
	19	0.8125	0.75	0.375
	20	0.65625	0.875	0.375
Immunodeficiencies	1	0.59375	1	0.375
	2	0.65625	0.75	0.375
	3	0.59375	0.875	0.375
	4	0.53125	0.5	0.625
	5	0.90625	0.375	0.125
	6	0.9375	0	0.125
	7	0.75	0.625	0.625
	8	0.65625	0.75	0.5
	9	0.90625	0.25	0.25
	10	0.84375	-0.25	0
	11	0.71875	0.5	0
	12	0.6875	0.5	0.5
	13	0.59375	0.375	-0.125
	14	0.78125	0.25	0.125
	15	0.5625	0.75	0.625
	16	0.5	0	-0.125
	17	0.53125	0.75	0.125
	18	0.4375	0.625	0
	19	0.78125	0.25	0.375
	20	0.40625	0.875	0.375
Sarcoidosis and Miscellaneous Rheumatologic Conditions	1	0.90625	0	-0.125
	2	0.9375	0.25	0.125
	3	0.71875	0.625	0
	4	0.65625	0.75	0.375
	5	0.90625	0.125	0.125
	6	0.21875	0.125	-0.25
	7	0.59375	0.5	0.5
	8	0.5625	0.375	0.625
	9	0.71875	0.375	0.25
	10	0.8125	0.375	-0.125
	11	0.5625	0.75	0
	12	0.90625	0.125	-0.125
	13	0.65625	0.125	-0.25
	14	0.96875	0.125	0.125
	15	0.90625	0.125	0.25
	16	0.84375	0.25	0.25
	17	0.71875	0.375	0.125
	18	0.875	0.125	0
	19	0.5625	0.25	0
	20	0.40625	0.5	0
Steroids	1	0.96875	0.125	0.125
	2	1	0	0
	3	0.84375	0.25	0.125
	4	0.96875	0.125	0.125
	5	0.71875	0.5	0.5
	6	0.78125	0.5	0.25
	7	0.9375	0.25	0.125
	8	0.8125	0.75	0.5
	9	0.53125	0.625	0.375
	10	0.75	0.375	0
	11	0.5625	0.25	0
	12	0.4375	0.5	0.5
	13	0.59375	0.625	0.375
	14	0.53125	0.75	0.375
	15	0.53125	0.625	0.5
	16	0.5	0.25	0.25
	17	0.75	0.5	0.25
	18	0.71875	0.375	0.25
	19	0.90625	0.25	0
	20	0.5	0.875	0.5
NSAIDs and Colchicine	1	1	0	0
	2	0.96875	0.125	0.125
	3	0.9375	0.125	0.125
	4	0.84375	0.375	0
	5	0.875	0.25	0
	6	0.71875	0.375	0.25
	7	0.5	0.5	-0.125
	8	0.46875	0.25	0
	9	0.5625	0.5	0.625
	10	0.78125	0.375	0.125
	11	0.875	0.125	0.125
	12	0.84375	0.25	0.25
	13	0.84375	0.375	0.375
	14	0.625	0.5	0.375
	15	0.53125	0.5	0.375
	16	0.75	0.5	0.25
	17	0.71875	0.25	0.375
	18	0.8125	0.375	0.125
	19	0.84375	0.375	0.25
	20	0.71875	0.75	0.75
Principles of Rheumatologic Disease Management	1	0.65625	0.5	0.125
	2	0.59375	0.375	0.25
	3	0.5625	0.5	0.125
	4	0.75	0.5	0.5
	5	0.9375	0.25	0.125
	6	0.875	0.25	0
	7	0.84375	0.25	0
	8	0.625	0.375	0.25
	9	0.90625	0.125	0
	10	0.46875	0.375	0
	11	0.71875	0.5	0.375
	12	0.875	0.25	0.125
	13	0.6875	0.25	0.375
	14	0.78125	0.375	0.25
	15	0.625	0.75	0.375
	16	0.4375	0.75	0.5
	17	0.71875	0.125	0.25
	18	0.78125	0.25	0.25
	19	0.84375	0.125	0.25
	20	0.9375	0.25	0.25
Methotrexate and Leflunomide	1	0.75	0	0.25
	2	0.8125	0.375	0
	3	0.84375	0.375	-0.125
	4	0.625	0.75	0.5
	5	0.78125	0.375	0.375
	6	0.96875	0.125	0.125
	7	0.59375	0.5	0.125
	8	0.96875	0.125	-0.125
	9	0.9375	0.125	0.125
	10	0.8125	0.25	0.375
	11	0.34375	0.5	0.25
	12	0.90625	0.125	0.25
	13	0.84375	0.5	0.25
	14	0.8125	0.5	0.375
	15	0.96875	0.125	0
	16	0.875	0.25	0.25
	17	0.71875	0	0
	18	0.59375	0.375	0.125
	19	0.6875	0.5	0.25
	20	0.75	0.625	0.375
Sulfasalazine and Hydroxychloroquine	1	0.53125	1	0.125
	2	0.875	0.25	0
	3	0.9375	0.125	-0.125
	4	0.6875	0.625	0.375
	5	0.90625	0.125	0
	6	0.46875	0.75	0.5
	7	0.6875	0.625	0.375
	8	0.8125	0.125	0.125
	9	0.53125	0.625	0.25
	10	0.5625	0.25	0.25
	11	0.75	0.75	0.125
	12	0.625	0.75	0.5
	13	0.875	0.125	0
	14	0.625	0.875	0.625
	15	0.75	0.75	0.125
	16	0.6875	0.5	0.375
	17	0.9375	0.125	0
	18	0.5625	0.5	0.625
	19	0.84375	0.25	0.25
	20	0.5625	0.5	0.375
	21	0.65625	0.5	0.25
	22	0.625	0.375	0.125
	23	0.75	0.375	0.375
	24	0.6875	0.5	0.25
	25	0.71875	0.375	0
Azathioprine and Mycophenolate Mofetil	1	0.46875	11.75	0.249023438
	2	0.6875	0.375	0.25
	3	0.8125	0.125	0.125
	4	0.46875	0.125	0.375
	5	0.96875	-0.125	0.125
	6	0.5625	0.125	0.5
	7	0.53125	0	0
	8	0.59375	0	0.5
	9	0.75	0	0.375
	10	0.875	0	0
	11	0.9375	0	0.25
	12	0.6875	0.375	0.375
	13	0.53125	-0.25	0.375
	14	0.5625	-0.125	0.625
	15	0.65625	0	0.375
	16	0.6875	0.25	0.375
	17	0.6875	0	0.125
	18	0.8125	0	0.375
	19	0.6875	0.125	0.5
	20	0.75	-0.125	0.25
Cyclophosphamide, Cyclosporine, and Tacrolimus	1	0.5625	0.375	0.5
	2	0.8125	0.25	0.375
	3	0.65625	0.375	0.125
	4	1	0	0
	5	0.5	0.75	0.25
	6	0.78125	0.375	0.25
	7	0.46875	0.75	0.25
	8	0.625	0.5	0.375
	9	0.53125	0.5	0.375
	10	0.90625	0	0
	11	0.9375	0	0.125
	12	0.46875	0.625	0.25
	13	0.9375	0.25	0.125
	14	0.71875	0.125	0.25
	15	0.4375	0.75	0.25
	16	0.53125	0.25	0.375
	17	0.8125	0.375	0.25
	18	0.84375	0.5	0.125
	19	0.46875	1	0.25
	20	0.9375	0.25	0.25
Urate-Lowering Therapy	1	0.875	0	0.25
	2	0.78125	-0.125	0.125
	3	0.75	0.125	0.375
	4	0.84375	0	0.375
	5	0.71875	0.125	0.375
	6	0.71875	0.25	0.125
	7	0.96875	0	0
	8	0.875	0	0.125
	9	0.71875	0.125	0
	10	0.71875	0.125	0.375
	11	0.96875	-0.125	0.125
	12	0.71875	0	0.375
	13	0.6875	0	0
	14	0.625	0	0.25
	15	1	0	0
	16	0.84375	0	0.25
	17	0.8125	-0.125	0
	18	0.5	0.125	0
	19	0.6875	0	-0.125
	20	0.9375	0	0
	21	0.96875	0.125	-0.125
Other Conventional DMARDs	1	0.59375	0.75	0
	2	0.78125	0	0
	3	0.59375	0.625	0.5
	4	0.53125	0.5	0.25
	5	0.90625	0.375	0.125
	6	0.84375	0.25	0.25
	7	0.625	0.5	0.375
	8	0.28125	0.375	0.25
	9	0.625	0.5	0.25
	10	0.78125	0.375	0
	11	0.90625	0.25	0.125
	12	0.40625	0.375	0.75
	13	0.71875	0.375	0.125
	14	0.8125	-0.125	0
	15	0.40625	0.625	0.5
	16	0.46875	0.875	0.5
	17	0.75	0.25	0.125
	18	0.71875	0.375	0
	19	0.59375	0.625	0
	20	0.5625	0.875	0.25
TNF Inhibitors	1	0.53125	0.5	0.125
	2	0.59375	0.25	0.25
	3	0.78125	0.5	0.375
	4	0.8125	0.375	0.5
	5	0.875	0.25	0.375
	6	0.46875	0.5	0.375
	7	0.71875	0.25	0
	8	0.78125	0.5	0.375
	9	0.96875	0.125	0.125
	10	0.65625	0.5	0.25
	11	0.90625	0.375	0.125
	12	0.75	0.5	0.25
	13	0.78125	0.375	0.25
	14	0.96875	0	0
	15	0.875	0.375	0.25
	16	0.5	0.25	-0.125
	17	0.8125	0.375	0.125
	18	0.875	0.375	0.25
	19	0.8125	0.5	0.25
	20	0.5	0.375	-0.125
Drugs Targeting IL1, IL12, IL17 and IL23	1	0.90625	0.25	0.125
	2	0.78125	0.375	0.375
	3	0.5625	0.25	0.125
	4	0.6875	0.75	0.25
	5	0.5	0.375	-0.125
	6	0.65625	0.625	0.125
	7	0.59375	0.625	0.375
	8	0.5	0.75	0.375
	9	0.9375	0.125	0
	10	0.84375	0.125	0
	11	0.5	0.5	0.25
	12	0.875	0.125	0
	13	0.96875	0.125	0.125
	14	0.8125	0.25	0
	15	0.75	0.75	0.5
	16	0.625	0.75	0.375
	17	0.625	0.75	0.75
	18	0.53125	0.375	0.875
	19	0.75	0.125	0.125
	20	0.53125	0	0.125
Rituximab, Belimumab,a nd Tocilizumab	1	1	0	0
	2	0.96875	0	-0.125
	3	0.625	0.5	0.375
	4	0.96875	0.125	0.125
	5	0.90625	0.125	-0.25
	6	0.625	0.125	0.375
	7	0.96875	0	0
	8	0.875	0.125	0.125
	9	0.96875	0.125	0.125
	10	0.84375	0.25	0.25
	11	0.46875	0.875	0
	12	0.8125	0.5	-0.125
	13	0.4375	0.375	0.375
	14	0.6875	0.25	0.5
	15	0.65625	0.5	0.25
	16	0.75	0.25	0.25
	17	0.375	0.625	0
	18	0.9375	0.25	0.25
	19	0.84375	0.375	0.25
	20	0.5	0.625	0.25
Abatacept, Apremilast, Mepolizumab, and Jakinibs	1	0.9375	0	0.125
	2	0.8125	0.25	0.125
	3	1	0	0
	4	0.65625	0.5	0.5
	5	0.71875	0.5	0.125
	6	0.96875	0.125	0.125
	7	0.6875	0.5	0.125
	8	0.5	0.875	0.125
	9	0.46875	0.375	-0.25
	10	0.5	0.375	-0.125
	11	0.375	0.25	0.125
	12	0.59375	0.375	0.375
	13	0.78125	0.5	0.125
	14	0.59375	0.75	0.625
	15	0.8125	0.375	0
	16	0.71875	0.25	0.625
	17	0.78125	0.5	-0.125
	18	0.5	0.375	0.375
	19	0.8125	0	0.375
	20	0.625	0.625	0.25
Metabolic and Crystalline Diseases	1	0.65625	0.125	0
	2	0.5625	0.625	0.375
	3	0.625	0.25	0.25
	4	0.5625	0.375	0.25
	5	0.8125	0.25	0.25
	6	0.75	0.25	0.125
	7	0.53125	1	0.25
	8	0.5	0.375	0.5
	9	0.375	0.25	0.25
	10	0.71875	0.5	0.375
	11	0.34375	0.5	0.25
	12	0.40625	0.75	0.625
	13	0.25	0.375	0.25
	14	0.28125	0.625	0.5
	15	0.28125	0.375	0.25
	16	0.4375	0.125	0.125
	17	0.3125	0.375	0.25
	18	0.375	0.625	0.25
	19	0.84375	0.5	0.25
	20	0.5625	0.625	0.125
	21	0.6875	0.375	0.25
Gout	1	0.875	0.125	0.125
	2	1	0	0
	3	0.53125	0.75	0.25
	4	0.28125	0.375	0.125
	5	0.46875	0.625	0.25
	6	0.46875	0.625	0.125
	7	0.5625	0.625	0.25
	8	0.875	0.375	0.375
	9	0.53125	0.5	0.25
	10	1	0	0
	11	0.875	0.375	0.25
	12	0.96875	0.125	0.125
	13	0.5625	0.75	0
	14	0.46875	0.375	0.375
	15	0.6875	0.25	0.125
	16	0.53125	0.5	0.5
	17	0.46875	0.875	0.375
	18	0.65625	0.375	0
	19	0.65625	0.625	0.375
	20	0.71875	0.25	0.375
Calcium Pyrophosphate Arthritis	1	0.75	0.375	0
	2	0.53125	0.625	0.375
	3	0.59375	0.625	0.25
	4	0.875	0.25	0
	5	0.40625	0.5	-0.25
	6	0.71875	0.25	-0.25
	7	0.6875	0.25	0.125
	8	0.40625	0.625	0.375
	9	0.65625	0	-0.125
	10	0.53125	0.5	0.25
	11	0.78125	0.5	0.25
	12	0.46875	0.625	0.375
	13	0.40625	0.5	0.125
	14	0.53125	0.375	0.375
	15	0.625	0.625	0.5
	16	0.84375	0.375	0.25
	17	0.9375	0.125	0.25
	18	0.40625	0	-0.125
	19	0.46875	0.875	0.25
	20	0.3125	0.375	0.125
Osteoarthritis	1	0.96875	0.125	0.125
	2	0.9375	0.125	0.25
	3	0.34375	0.125	0.25
	4	0.8125	0.5	0.5
	5	0.71875	0.25	0.375
	6	0.96875	0.125	0.125
	7	0.625	0.375	0.25
	8	0.625	0.75	0.5
	9	0.53125	0.5	0.25
	10	0.3125	0.625	0.25
	11	0.46875	0.375	-0.125
	12	0.53125	0.625	0.125
	13	0.4375	0.5	0.5
	14	0.375	0.25	0.125
	15	0.4375	0.75	0
	16	0.125	0.125	0
	17	0.6875	0.25	0.125
	18	0.375	0.5	0.375
	19	0.40625	0.75	0.125
	20	0.3125	0.125	0.25
Osteoporosis	1	0.59375	0.75	0.75
	2	0.75	0.5	0
	3	0.6875	0.625	0.25
	4	0.9375	0.125	0.125
	5	0.78125	-0.125	0.125
	6	0.59375	0.125	0.25
	7	0.75	0.375	0.25
	8	0.75	0.25	0.125
	9	0.6875	0	0.375
	10	0.59375	0.5	-0.25
	11	0.375	0.625	0.375
	12	0.59375	0.5	0.25
	13	0.65625	0.25	0.125
	14	0.53125	0.5	0
	15	0.4375	0.75	0.375
	16	0.625	0.5	0.25
	17	0.6875	0.5	0.5
	18	0.78125	0.375	0.375
	19	0.625	0.75	0.625
	20	0.71875	0.625	0.375
	21	0.6875	0.5	0.125
	22	0.5	0.125	0
	23	0.71875	0.25	-0.375
Paget Disease, Osteonecrosis, Osteomalacia, and HCTDs	1	0.28125	0.5	0
	2	0.71875	0	-0.125
	3	0.625	0.125	0.125
	4	0.46875	0	0.125
	5	0.84375	0	0.375
	6	0.59375	0.25	0.125
	7	0.6875	0.25	-0.125
	8	0.625	-0.25	0.125
	9	0.53125	0	0
	10	0.53125	-0.125	0.5
	11	0.59375	0.25	0
	12	0.625	0	0.375
	13	0.5625	0.25	0.125
	14	0.625	0	-0.125
	15	0.65625	0.125	0.125
	16	0.5	0.125	0.125
	17	0.71875	0.125	-0.375
	18	0.6875	0.125	-0.25
	19	0.4375	0.125	0.375
	20	0.125	0	0.125
	21	0.8125	-0.125	0.25
	22	0.46875	-0.375	0
	23	0.65625	0.125	0.625
	24	0.5	0	0.125
Ankylosing Spondylitis	1	0.96875	0.125	0.125
	2	1	0	0
	3	0.75	0.5	0.25
	4	0.65625	0.75	0.25
	5	0.71875	0.25	0
	6	0.8125	0.375	0.125
	7	0.625	0.75	0.375
	8	0.6875	0.5	0.375
	9	0.78125	0.125	0.375
	10	0.78125	0	-0.125
	11	0.65625	0.75	0.875
	12	0.625	0.625	0.25
	13	0.875	0.125	0.125
	14	0.65625	0.375	0.125
	15	0.65625	0.25	0
	16	0.6875	0.5	0.125
	17	0.6875	0.625	0.5
	18	0.90625	0.125	0
	19	0.59375	0.25	0.25
	20	0.46875	0.375	0.125
Reactive Arthritis	1	0.75	0.125	0.25
	2	0.6875	0.875	0.375
	3	0.71875	0.375	0.25
	4	0.625	0.375	0.25
	5	0.65625	0.75	0.5
	6	0.96875	0.125	0
	7	0.28125	0.625	0.25
	8	0.46875	0.875	0.5
	9	0.46875	0.25	0.25
	10	0.71875	0.625	0.5
	11	0.46875	0.5	0.25
	12	0.3125	0.375	0
	13	0.96875	0.125	0
	14	0.5	0.125	0.25
	15	0.53125	0.25	0.125
	16	0.4375	0.625	0.375
	17	0.78125	0.25	0
	18	0.5	0.625	0.375
	19	0.59375	0.125	0.375
	20	0.84375	0.375	0.25
Psoriatic Arthritis	1	0.96875	0	0
	2	0.75	0.25	0.375
	3	0.65625	0.75	0.125
	4	0.71875	0.25	0.5
	5	0.5	0.5	0.5
	6	0.9375	0	0.25
	7	0.8125	0.125	0.125
	8	0.5	0.5	-0.125
	9	0.75	0.25	-0.25
	10	0.4375	0.5	0.375
	11	0.40625	0.75	0.25
	12	0.375	0.25	0.375
	13	0.46875	0.5	0.25
	14	0.40625	0.625	0
	15	0.53125	0.375	0.375
	16	0.21875	0.25	0.125
	17	0.46875	0.125	0.125
	18	0.59375	0.25	0.375
	19	0.5	0.625	0
	20	0.8125	0.5	0.375
IBD-associated Arthritis	1	0.625	0.5	0.5
	2	0.46875	0.625	0.375
	3	0.46875	0.375	0.375
	4	0.625	0.375	0.25
	5	0.46875	0.375	0.625
	6	0.53125	0.5	0.5
	7	0.4375	0.375	0.25
	8	0.6875	0	-0.125
	9	0.78125	0.25	-0.125
	10	0.5	0.625	0.125
	11	0.46875	0.125	0.375
	12	0.4375	0.5	0.125
	13	0.59375	0.625	0.125
	14	0.5	0.625	0.375
	15	0.40625	0.75	0.5
	16	0.625	0.125	0.125
	17	0.625	0.5	0.375
	18	0.78125	0	0
	19	0.96875	0.125	0.125
	20	0.5	0.625	0.125
RA: Pathogenesis, Prognosis, and Epidemiology	1	0.625	0.375	0.5
	2	0.53125	0.75	0.5
	3	0.75	0.5	0.375
	4	0.875	0.25	0.125
	5	0.8125	0.125	0.25
	6	0.875	0	0.25
	7	0.5	0.25	0.375
	8	0.375	0.125	0.375
	9	0.59375	0.5	0.25
	10	0.53125	0.75	0.375
	11	0.53125	0.25	-0.25
	12	0.28125	0.75	0.25
	13	0.6875	0.625	0.375
	14	0.59375	-0.125	0
	15	0.5	0.25	0.25
	16	0.46875	0.625	0.375
	17	0.46875	0.375	0
	18	0.84375	0.25	0
	19	0.34375	0.625	0.5
	20	0.4375	0.375	0.5
RA: Diagnosis and Management Strategies	1	0.96875	0.125	0
	2	0.53125	0.5	0.5
	3	0.90625	0.25	0.25
	4	0.625	0.125	0.375
	5	0.375	0.25	0.125
	6	0.6875	0.5	0.25
	7	0.3125	0.375	-0.125
	8	0.65625	0.125	-0.375
	9	0.4375	0.625	0.375
	10	0.5625	0.75	0.5
	11	0.53125	0.375	0.125
	12	0.46875	0.5	0.125
	13	0.625	0.5	0
	14	0.5625	0.125	0.125
	15	0.71875	0.5	0.375
	16	0.53125	0.875	0.5
	17	0.5	0.375	0.125
	18	0.4375	0.5	0.625
	19	0.75	0.25	0.25
	20	0.46875	0.125	-0.125
RA: Extra-articular Manifestations	1	0.71875	0.375	0.125
	2	0.84375	0.125	0.375
	3	0.5	0.375	0.25
	4	0.65625	0.125	0.125
	5	0.8125	0.375	0.5
	6	0.5625	0.25	0.375
	7	0.25	0.625	0.375
	8	1	0	0
	9	0.78125	0.625	0.375
	10	0.75	0.5	0.5
	11	0.625	0.375	0
	12	0.5	0.625	0.5
	13	0.5	0.75	0.25
	14	0.4375	0.25	-0.375
	15	0.40625	0.5	0.125
	16	0.53125	0	-0.125
	17	0.53125	0.75	0.625
	18	0.25	0.5	0.25
	19	0.59375	0.375	0
	20	0.625	0.625	0.375
SLE: Pathogenesis, Diagnosis, and Epidemiology	1	0.4375	0.75	0.25
	2	0.34375	0.875	0.125
	3	0.46875	0.625	0.375
	4	0.6875	0	-0.125
	5	0.59375	0	0
	6	0.90625	0.25	0
	7	0.46875	0.625	0.375
	8	0.5	-0.125	0.25
	9	0.78125	0.125	-0.125
	10	0.4375	0.5	0.375
	11	0.375	0.375	0
	12	0.96875	0.125	0
	13	0.46875	0.375	0
	14	0.6875	0.375	0.25
	15	0.65625	0.375	0.5
	16	0.5	0.875	0.375
	17	0.65625	0.375	-0.125
	18	0.875	0.375	0.375
	19	0.3125	0.75	0.375
	20	0.28125	0.625	0.75
Cutaneous Manifestations of Lupus	1	0.59375	0.25	0.125
	2	0.375	0.25	0.5
	3	0.34375	0.5	0.125
	4	0.53125	0.5	0
	5	0.8125	0.375	0.375
	6	0.9375	0.25	0.125
	7	0.53125	0	0.25
	8	0.78125	0.625	0.375
	9	0.625	0.625	0.5
	10	0.375	0.375	0
	11	0.71875	0.375	0.25
	12	0.40625	0.625	0.375
	13	0.4375	0.625	0.125
	14	0.46875	0.25	0.25
	15	0.6875	0.375	0.25
	16	0.71875	0.5	0
	17	0.53125	0.5	0.25
	18	0.625	0.5	0.375
	19	0.3125	0.25	0.5
	20	0.46875	0.375	0.375
	21	0.375	0.5	0.375
Lupus Nephritis	1	0.65625	0.125	0
	2	0.53125	0.5	0.5
	3	0.625	0.75	0.375
	4	0.28125	0.125	0.25
	5	0.65625	0.625	0.5
	6	0.375	0.75	0.5
	7	0.625	0.75	0.25
	8	0.6875	0.5	0.125
	9	0.875	0.375	0.125
	10	0.34375	0.25	0.25
	11	0.53125	0.375	0.125
	12	0.40625	0.25	0.125
	13	0.71875	0.5	0.125
	14	0.40625	0.25	0
	15	0.5	-0.125	0.125
	16	0.71875	0.25	-0.125
	17	0.53125	0.375	0.375
	18	0.59375	0.5	0.25
	19	0.5625	0.625	0.25
	20	0.71875	0.75	0.625
APLS and SLE in Reproductive Health	1	0.6875	0.375	0
	2	0.65625	0.375	0.5
	3	0.34375	0.375	0.5
	4	0.5	0.5	0.25
	5	0.5	0.25	0.125
	6	0.28125	0.125	0.125
	7	0.71875	0.25	-0.125
	8	0.34375	0.625	0.125
	9	0.53125	0.5	0.375
	10	0.5625	0.5	0.25
	11	0.53125	0.625	0.375
	12	0.53125	0.625	0.125
	13	0.46875	0.125	0.125
	14	0.5	0.375	0.25
	15	0.8125	0.375	0.25
	16	0.5	0.375	0.375
	17	0.375	0.5	0.5
	18	0.625	0.25	-0.125
	19	0.65625	0.125	0
	20	0.65625	0.375	0.375
Systemic Sclerosis: Pathogenesis, Diagnosis, and Epidemiology	1	0.875	0.375	0.375
	2	0.4375	0.75	0.125
	3	0.25	0.375	-0.125
	4	0.46875	0.375	0.25
	5	0.40625	0.75	0.375
	6	1	0	0
	7	0.53125	0.25	0.125
	8	0.40625	0.375	0.125
	9	0.5625	0.5	0
	10	0.5625	0.125	0.375
	11	0.40625	0.375	0.625
	12	0.5	0.625	0.25
	13	0.25	0	0.125
	14	0.375	0.375	0.625
	15	0.5	0.375	0.25
	16	0.5	0.375	0.75
	17	0.3125	0.375	0.375
	18	0.375	0.375	0.125
	19	0.4375	0.625	0.375
	20	0.34375	0.5	0.125
	21	0.59375	0.125	0.375
	22	0.59375	0.125	-0.125
Visceral Manifestations of Systemic Sclerosis	1	0.71875	0.25	0.25
	2	0.375	0.375	0.625
	3	0.46875	0.75	0.625
	4	0.28125	0.5	0.125
	5	0.3125	0.375	0
	6	0.4375	0.25	0.25
	7	0.40625	0.375	0.125
	8	0.25	0.5	0.5
	9	0.625	0.5	0.25
	10	0.78125	0.625	0.375
	11	0.28125	0.25	-0.125
	12	0.25	0.125	0.125
	13	0.25	0.75	0.25
	14	0.28125	0.5	0.375
	15	0.3125	0.375	0.375
	16	0.375	0.375	0.375
	17	0.28125	0.625	0.375
	18	0.3125	0.125	0.125
	19	0.65625	0.25	0.25
	20	0.375	0.625	0.75
Localized Scleroderma and Ranaud Phenomenon	1	0.3125	0.625	0.625
	2	0.375	0.375	0.125
	3	0.3125	0.5	0.375
	4	0.15625	0.25	0.25
	5	0.78125	0.5	0.25
	6	0.21875	0.5	0.375
	7	0.375	0.75	0.5
	8	0.40625	0.375	0.5
	9	0.6875	0.125	0.375
	10	0.09375	0.125	0.25
	11	0.6875	0.75	0.5
	12	0.21875	0.25	0
	13	0.40625	0.375	0.125
	14	0.21875	0.375	0.5
	15	0.375	0.5	0.625
	16	0.40625	0.5	0.125
	17	0.28125	0.375	0.125
	18	0.25	0.125	0
	19	0.34375	0.25	0.25
	20	0.15625	0.125	0
Scleroderma Mimics	1	0.15625	-0.125	0
	2	0.40625	0.5	0.375
	3	0.25	0.625	0.5
	4	0.65625	0.625	0.25
	5	0.25	0.25	0.125
	6	0.1875	0.25	0.375
	7	0.28125	0.375	0.25
	8	0.375	0.625	0.375
	9	0.1875	0.125	-0.125
	10	0.4375	0.5	0.375
	11	0.46875	0.375	0.25
	12	0.34375	0.375	0.125
	13	0.40625	0.5	0.5
	14	0.625	0.75	0.25
	15	0.3125	0.5	0.375
	16	0.53125	0	0.375
	17	0.875	0.5	0.25
	18	0.4375	0.375	0.5
	19	0.40625	0.125	0.25
	20	0.25	0.375	0
	21	0.46875	0.5	0.375
	22	0.375	0.125	-0.125
	23	0.5625	0.25	0.125
	24	0.25	-0.125	0.125
Inflammatory Myositis: Prognosis and Treatment	1	0.53125	0.75	0.25
	2	0.3125	0.375	0.375
	3	0.25	0.25	0.125
	4	0.25	0.25	0.375
	5	0.1875	0.25	-0.25
	6	0.625	0.625	0.625
	7	0.28125	-0.125	0.125
	8	0.3125	0.75	0.5
	9	0.625	0.5	0.375
	10	0.28125	0.375	0.125
	11	0.34375	0.25	0.25
	12	0.34375	0.625	0.375
	13	0.5625	0.375	0.375
	14	0.53125	0.75	0.5
	15	0.28125	0.375	0.375
	16	0.40625	0.75	0.125
	17	0.53125	0.25	0.125
	18	0.71875	0.25	0.125
	19	0.21875	0.125	0
	20	0.5	0.125	0.125
	21	0.34375	0.375	0.125
Inflammatory Myositis: Pathophysiology and Diagnosis	1	0.84375	0.5	0.375
	2	0.28125	0.25	-0.25
	3	0.3125	0.125	0
	4	0.34375	0.5	0.125
	5	0.46875	0.875	0.5
	6	0.59375	0.625	0
	7	0.5	0	0.5
	8	0.9375	0.25	0.125
	9	0.5	0.625	0.5
	10	0.3125	-0.125	0.125
	11	0.40625	-0.125	0
	12	0.46875	0.125	0.5
	13	0.25	0.5	0.375
	14	0.625	0.5	-0.25
	15	0.4375	0.375	0.125
	16	0.3125	0.125	0.25
	17	0.40625	0.75	0.625
	18	0.5625	0.5	0.375
	19	0.53125	0.5	0
	20	0.5625	0.25	0.25
	21	0.15625	0.125	0
	22	0.28125	0.125	0.25
	23	0.5625	0.375	0.375
Myositis Mimics	1	0.5625	0.375	0.25
	2	0.4375	0.5	0.25
	3	0.28125	0.75	0.375
	4	0.28125	0.125	-0.125
	5	0.125	0.125	0
	6	0.40625	0.625	0.25
	7	0.34375	0.25	0.125
	8	0.46875	0	0
	9	0.375	0.25	0.375
	10	0.34375	0.25	0
	11	0.53125	0.5	0.25
	12	0.375	0.875	0.25
	13	0.375	0.5	0
	14	0.28125	0.125	0
	15	0.375	0.5	0.375
	16	0.5	0.375	0.375
	17	0.375	0.5	0.125
	18	0.78125	0.5	0.25
	19	0.53125	0.625	0.375
	20	0.5625	0.125	0.125
Sjogren Syndrome	1	0.65625	0.125	-0.125
	2	0.28125	0.25	0.375
	3	0.5	0.625	0.5
	4	0.46875	0.25	-0.125
	5	0.46875	0.25	-0.125
	6	0.46875	0.5	0
	7	0.25	-0.125	0
	8	0.3125	0.125	0.25
	9	0.3125	0.375	0.375
	10	1	0	0
	11	0.25	0.25	0
	12	0.625	0.875	0.625
	13	0.40625	1	0.5
	14	0.71875	0.625	0.375
	15	0.75	0.625	0.375
	16	0.65625	0.75	0.75
	17	0.75	0.125	0.125
	18	0.65625	0.625	0.25
	19	0.28125	0.125	0.375
	20	0.40625	0.75	0.125
MCTD, UCTD, and Overlap Syndromes	1	0.78125	0.375	0.25
	2	0.96875	0.125	0.125
	3	0.59375	0.375	0
	4	0.375	0.5	0.25
	5	0.5	0.375	0.25
	6	0.5625	0.5	0.25
	7	0.4375	0.125	-0.125
	8	0.4375	0.375	0.25
	9	0.59375	0.625	0
	10	0.5	0.375	0.125
	11	0.40625	0.375	-0.125
	12	0.53125	0.125	-0.125
	13	0.5625	0.75	0.25
	14	0.5625	0.5	0.25
	15	0.4375	0.375	-0.125
	16	0.375	0.25	0
	17	0.4375	0.375	0.125
	18	0.46875	0.375	0.25
	19	0.21875	0.25	0.125
	20	0.625	0.375	-0.25
Giant Cell Arteritis	1	0.625	0.5	0.125
	2	0.625	0.25	0.25
	3	0.5625	0.25	-0.125
	4	0.4375	0.375	0.5
	5	0.59375	0.125	0.25
	6	0.46875	0.625	0.5
	7	0.96875	0	0.125
	8	0.4375	0.375	0.375
	9	0.4375	0.375	0.375
	10	0.5	0.625	-0.125
	11	0.5625	0.5	0.375
	12	0.4375	0.375	0.125
	13	0.9375	0.25	0.125
	14	0.875	0.375	0.375
	15	0.625	0.375	0.375
	16	0.46875	0.375	0.25
	17	0.53125	0.625	0.625
	18	0.34375	0.25	0.125
	19	0.59375	0.25	0
	20	0.15625	0.375	0.125
PMR & Takayasu Arteritis	1	0.5625	0.375	0.5
	2	0.5	0.5	0.125
	3	0.53125	0.25	0
	4	0.4375	0.25	0.375
	5	0.9375	0.125	-0.125
	6	0.34375	-0.125	-0.125
	7	0.375	0.25	0
	8	0.34375	0.5	0.25
	9	0.46875	0.625	0.75
	10	0.4375	0.875	0.125
	11	0.4375	0.75	0.5
	12	0.5	0.125	-0.125
	13	0.34375	0.25	0.5
	14	0.6875	0.375	0.375
	15	0.3125	0.5	0.25
	16	0.21875	0.25	0.125
	17	0.75	0.25	0.375
	18	0.3125	0.25	0
	19	0.46875	0.5	0.75
	20	0.375	0.5	0.375
	21	0.4375	0.125	0
	22	0.28125	0.375	0
	23	0.6875	0.375	0.25
Polyarteritis Nodosa	1	0.71875	0.125	0.375
	2	0.5625	0.625	0.375
	3	0.53125	0.625	0.125
	4	0.34375	0.5	0.375
	5	0.5	0.625	0.5
	6	0.375	0.625	0.375
	7	0.3125	0	0.25
	8	0.1875	0.25	0
	9	0.40625	0.125	-0.25
	10	0.8125	0.375	0.25
	11	0.375	0.125	0
	12	0.375	0.5	0.375
	13	0.21875	0.25	0.25
	14	0.34375	0.75	0.625
	15	0.4375	0.375	0.375
	16	0.4375	0.125	0.25
	17	0.46875	0.375	0.375
	18	0.46875	0.75	0.5
	19	0.5	0.25	0.125
	20	0.46875	0.25	0.125
Variable Vessel Vasculitides and Thromboangiitis Obliterans	1	0.375	0.75	0
	2	0.5	0.125	0.5
	3	0.28125	0.5	0.125
	4	0.46875	0.375	-0.25
	5	0.40625	0	-0.125
	6	0.59375	0.625	0.375
	7	0.1875	0	0.125
	8	0.34375	-0.125	0
	9	0.3125	0	-0.125
	10	0.4375	0.25	-0.125
	11	0.5625	0.75	-0.125
	12	0.5	0.625	0.125
	13	0.5625	0.75	0.25
	14	0.65625	0.625	0.125
	15	0.375	0.75	0
	16	0.3125	0.625	-0.25
	17	0.5625	-0.125	0
	18	0.375	0.625	0
	19	0.40625	0.375	0.125
	20	0.21875	0.375	0.125
Vasculitis Mimics	1	1	0	0
	2	0.6875	0.375	0.25
	3	0.65625	0.75	-0.125
	4	0.5625	0	0.125
	5	0.65625	0.375	0
	6	0.4375	0.5	-0.125
	7	0.6875	0.5	0.25
	8	0.28125	0.25	0
	9	0.875	0.25	0.375
	10	0.25	0.375	-0.125
	11	0.40625	0.625	0.75
	12	0.625	0.5	0
	13	0.96875	0	0
	14	0.15625	0.25	0.125
	15	0.3125	0.625	-0.125
	16	0.4375	0.25	-0.125
	17	0.4375	0.5	0.375
	18	0.59375	0.25	0.25
	19	0.4375	-0.125	0.25
	20	0.21875	0.375	-0.25
Primary CNS Vasculitis	1	0.375	0.25	0.125
	2	0.4375	0.75	0.375
	3	0.78125	0.5	0.125
	4	0.53125	0.625	0.625
	5	0.4375	0.25	0
	6	0.65625	0.5	0.375
	7	0.375	0.375	0
	8	0.65625	0.375	0
	9	0.4375	0.375	0.25
	10	0.3125	0	0.5
	11	0.4375	0.25	0.25
	12	0.71875	0	-0.125
	13	0.75	0.375	0.375
	14	0.5	0.625	0.5
	15	0.375	0.125	0
	16	0.3125	0.5	0.375
	17	0.46875	0.75	0.125
	18	0.90625	0.125	0.25
	19	0.3125	0.5	0.5
	20	0.375	0.375	0.625
ANCA-Associated Vasculitis: Diagnosis & Clinical Presentation	1	0.75	0.5	0.625
	2	0.46875	0.375	0.125
	3	0.15625	0.25	-0.125
	4	0.4375	0.5	0.375
	5	0.6875	0.125	0.125
	6	0.4375	0.375	0
	7	0.65625	0.25	0.25
	8	0.34375	0.625	0.625
	9	0.4375	0.125	0.375
	10	0.28125	0.25	0.125
	11	0.28125	-0.125	-0.125
	12	0.3125	0.625	0.25
	13	0.34375	0.125	0.375
	14	0.46875	0.5	0.25
	15	0.4375	0.625	0.375
	16	0.625	0.5	0.375
	17	0.71875	0.625	0.625
	18	0.40625	0.25	0.375
	19	0.28125	0.5	0.25
	20	0.25	0.375	0.25
ANCA-Associated Vasculitis: Associated Diseases, Prognosis & Treatment	1	0.75	0.375	0.25
	2	0.15625	0	0.125
	3	0.375	0.5	0.375
	4	0.3125	0.375	0.125
	5	0.5	0.375	0.5
	6	0.15625	0.375	0.125
	7	0.21875	0.375	-0.125
	8	0.34375	0.375	-0.125
	9	0.8125	0.625	0.25
	10	0.46875	0.625	0.625
	11	0.3125	0.625	0.25
	12	0.28125	0.5	0.5
	13	0.4375	0.375	0.5
	14	0.25	0.375	0.125
	15	0.46875	0.625	0.25
	16	0.28125	0.375	0.125
	17	0.3125	0	0
	18	0.15625	0.5	0
	19	0.21875	0.375	0.25
	20	0.4375	0.5	0.25
Cutaneous Small Vessel Vasculitides (except IgA Vasculitis)	1	0.15625	0	0
	2	0.46875	0.125	0.125
	3	0.53125	0.75	-0.125
	4	0.53125	0.375	-0.125
	5	0.4375	0.5	-0.125
	6	0.8125	0.25	0
	7	0.375	0.125	0.25
	8	0.25	0.375	0.25
	9	0.40625	0.75	0.5
	10	0.3125	0.625	0
	11	0.15625	0.375	-0.125
	12	0.3125	0.625	-0.375
	13	0.46875	0.375	0.5
	14	0.40625	0.125	0.25
	15	0.125	0.25	0
	16	0.53125	0.25	-0.375
	17	0.53125	0.75	0
	18	0.5	1	0.125
	19	0.25	0.375	0.25
	20	0.53125	0.5	0.125
IgA Vasculitis	1	0.1875	-0.125	0
	2	0.25	0.125	0.125
	3	0.34375	0.625	0.5
	4	0.40625	0.375	0.125
	5	0.375	0.5	-0.125
	6	0.5	0.75	0.25
	7	0.875	0.375	0.375
	8	0.84375	0.125	0.125
	9	0.21875	0.25	0.25
	10	0.1875	0	0
	11	0.5625	0.625	0.5
	12	0.3125	0	0.125
	13	0.4375	0.5	0.25
	14	0.5	0.5	0
	15	0.59375	0.625	0.25
	16	0.4375	0.25	0
	17	0.25	0.625	0.125
	18	0.40625	0.5	0.25
	19	0.40625	0.5	0.25
	20	0.15625	0.375	0.25
Cryoglobulinemia and Related Diseases	1	0.875	0.5	0.125
	2	0.125	0.125	0.25
	3	0.3125	0.5	0.25
	4	0.1875	0.625	0
	5	0.34375	0.375	0
	6	0.34375	0.25	0
	7	0.71875	0.625	0
	8	0.375	0.75	0
	9	0.34375	0.25	-0.25
	10	0.46875	0.375	0.125
	11	1	0	0
	12	0.3125	0.375	0.125
	13	0.3125	0.75	0.25
	14	0.25	0.125	0.25
	15	0.5625	0.75	0.125
	16	0.46875	0.125	0.375
	17	0.53125	0.625	0
	18	0.34375	0.25	-0.125
	19	0.21875	0.5	-0.125
	20	0.53125	0.5	-0.375
Relapsing Polychondritis	1	0.34375	0.25	0.25
	2	0.1875	0.375	0.375
	3	0.125	0.125	0
	4	0.375	0.625	0.125
	5	0.375	0.375	-0.125
	6	0.3125	0.75	0.25
	7	0.59375	0.375	-0.125
	8	0.375	0.5	0.75
	9	0.4375	0.625	0.375
	10	0.28125	0.5	0.25
	11	0.40625	0.375	0
	12	0.46875	0.25	0
	13	0.46875	0.375	0.125
	14	0.5625	0.75	0.125
	15	0.375	0.375	-0.125
	16	0.375	0.25	-0.375
	17	0.40625	0.125	0.375
	18	0.4375	0.5	0.25
	19	0.6875	0.625	0.125
	20	0.5	0.625	0.125
IgG4-Related Disease	1	0.84375	0.625	0.5
	2	0.46875	0.625	0.25
	3	0.40625	0.25	-0.375
	4	0.59375	0.375	0.5
	5	0.1875	0.125	0.125
	6	0.625	0.5	0.25
	7	0.65625	0.375	0.5
	8	0.9375	0.25	0.25
	9	0.40625	0.625	0
	10	0.5625	0.625	0.125
	11	0.46875	0.625	0.375
	12	0.65625	0.5	0.125
	13	0.75	0.5	0.5
	14	0.46875	0.5	0.125
	15	0.46875	0.125	0.125
	16	0.46875	0.375	0.25
	17	0.5	-0.125	0
	18	0.53125	0.25	0
	19	0.34375	0.125	0
	20	0.40625	0.25	0.125
	21	0.8125	0.25	0.25
Juvenile Idiopathic Arthritis	1	0.3125	0.875	0.125
	2	0.75	0.125	0
	3	0.46875	0.625	0
	4	0.5	0.375	0
	5	0.3125	0.5	-0.125
	6	0.46875	0.625	-0.125
	7	0.90625	0.125	-0.125
	8	0.28125	0.625	-0.125
	9	0.375	0.125	0
	10	0.375	0.5	-0.25
	11	0.21875	0.5	0.25
	12	0.65625	0.25	0
	13	0.75	0.625	0.25
	14	0.5625	0.5	0.25
	15	0.65625	0.125	0.25
	16	0.5	0.75	0.5
	17	0.46875	-0.25	0.25
	18	0.5	0.625	0.375
	19	0.40625	0.5	-0.25
	20	0.40625	0.75	0.25
Kawasaki Disease	1	0.40625	0.375	-0.125
	2	0.21875	0.25	0.125
	3	0.4375	0.625	0.375
	4	0.3125	0.875	0
	5	0.71875	0.375	0.125
	6	0.53125	0.5	0.25
	7	0.9375	0.125	0.125
	8	0.5	0.5	-0.125
	9	0.1875	0.5	0.125
	10	0.6875	0.5	-0.125
	11	0.28125	0.625	0.25
	12	0.875	0.375	0.125
	13	0.5	0.625	0.125
	14	0.5	0.5	-0.375
	15	0.5	0.375	0.25
	16	0.40625	0.125	0.375
	17	0.3125	0.125	0.125
	18	0.4375	0.75	0.125
	19	0.15625	0.5	0
	20	0.59375	0.5	0.25
Periodic Fever Syndromes	1	0.4375	0.75	0.25
	2	0.3125	0.5	0.125
	3	0.46875	0.5	-0.125
	4	0.40625	0.125	0.125
	5	0.25	0	-0.375
	6	0.375	0.125	-0.125
	7	0.46875	0.625	0.25
	8	0.4375	-0.125	0.25
	9	0.1875	0.375	-0.25
	10	0.40625	0.75	0
	11	0.28125	0.625	0
	12	0.3125	0.375	0.25
	13	0.28125	0.125	0.125
	14	0.3125	0.5	0
	15	0.5625	0.375	-0.125
	16	0.5	0.75	0.375
	17	0.15625	0.375	0
	18	0.28125	0.5	0.25
	19	0.65625	0.375	0.25
	20	0.53125	0.375	0.25
Autoinflammatory Syndromes	1	0.15625	0	0
	2	0.15625	0.25	0.125
	3	0.15625	0.125	-0.25
	4	0.375	0.125	0.125
	5	0.1875	-0.125	-0.125
	6	0.78125	0	0
	7	0.53125	0.125	0.125
	8	0.25	0.125	0
	9	1	0	0
	10	0.46875	0.25	-0.125
	11	0.34375	0	0.125
	12	0.28125	0.25	0
	13	0.34375	0.375	0.25
	14	0.09375	0.125	0
	15	0.59375	-0.125	0
	16	0.1875	0	0.25
	17	1	0	0
	18	0.21875	0.25	-0.125
	19	0.21875	0	0.375
	20	0.3125	-0.125	-0.125
Septic Arthritis & Osteomyelitis	1	0.9375	-0.125	-0.125
	2	0.25	0	0
	3	0.25	0	0.25
	4	0.21875	0.625	0.375
	5	0.15625	0.25	-0.125
	6	0.25	0.375	0.5
	7	0.71875	0.625	0.25
	8	0.25	0.375	0.375
	9	0.4375	0.5	0.5
	10	0.53125	0.25	0.25
	11	0.21875	0.125	-0.125
	12	0.65625	0.625	0
	13	0.34375	0.625	0.25
	14	0.34375	0.625	0.125
	15	0.40625	0.25	0
	16	0.5	0.25	0.125
	17	0.3125	0.375	0.125
	18	0.90625	0.25	0.125
	19	0.5625	0.375	0.25
	20	0.625	0.375	0.75
Lyme Disease	1	0.9375	0.125	-0.125
	2	0.875	0.125	-0.125
	3	0.59375	0.25	0.625
	4	0.28125	0.625	-0.25
	5	0.4375	0.375	0.25
	6	0.5	0.375	0.125
	7	0.53125	0.625	-0.125
	8	0.34375	0.5	-0.25
	9	0.34375	0.625	0.25
	10	0.53125	0.5	0.25
	11	0.25	0.375	0.125
	12	0.59375	0.375	0
	13	0.84375	0.125	0
	14	0.65625	0.25	-0.125
	15	0.3125	0.375	0.125
	16	0.4375	0.875	0
	17	0.21875	0.5	0.125
	18	0.3125	0.5	0
	19	0.375	0.375	0
	20	0.125	0.375	0.125
Whipple Disease	1	0.3125	0.625	0.125
	2	0.15625	0.375	0.25
	3	0.4375	0.75	0.375
	4	0.4375	0.75	0.125
	5	0.5	0.25	0.25
	6	0.46875	0.75	0.375
	7	0.375	0.375	0
	8	0.34375	0.625	0.375
	9	0.1875	-0.25	-0.125
	10	0.1875	0.125	0
	11	0.34375	0.5	0.125
	12	0.3125	0.25	0.125
	13	0.25	0.375	0.375
	14	0.375	0.125	0.25
	15	0.3125	0.75	0.5
	16	0.5	0.375	0.25
	17	0.375	0.125	0.125
	18	0.1875	0.125	0.125
	19	0.25	0.625	0.625
	20	0.21875	0.5	0.25
Rheumatic Fever & Post-Infectious Reactive Arthritis	1	0.59375	0.75	0.125
	2	0.1875	0.5	-0.125
	3	0.1875	0.125	0.125
	4	0.25	0.375	0.375
	5	0.53125	0.625	0
	6	0.5	0.5	0.125
	7	0.3125	0.375	-0.125
	8	0.4375	0.5	0.25
	9	0.75	0.5	-0.125
	10	0.28125	0	0.25
	11	0.25	0.125	0.125
	12	0.46875	0.5	0.25
	13	0.5	0.5	0
	14	0.21875	0.375	0.25
	15	0.28125	0.125	-0.125
	16	0.25	0.625	0.375
	17	0.46875	0.25	0.375
	18	0.71875	0.625	0
	19	0.4375	0.125	0
	20	0.28125	0.375	-0.125
Disseminated Gonococcemia	1	0.40625	0.25	-0.125
	2	0.5625	0.625	0.25
	3	0.21875	0.125	0
	4	0.34375	0.375	0.125
	5	0.25	0.25	0.125
	6	0.4375	0.125	-0.125
	7	0.46875	0.5	0.375
	8	0.5625	0.625	0.25
	9	0.78125	0.5	0.125
	10	0.28125	0.625	0.5
	11	0.625	0.5	0.25
	12	0.25	0.5	0.125
	13	0.3125	0.25	-0.125
	14	0.5625	0.375	0.25
	15	0.375	0.75	0.375
	16	0.34375	0.625	0.375
	17	0.09375	-0.125	0.125
	18	0.375	0.25	0.125
	19	0.21875	0.375	0.25
	20	0.21875	0.25	0.25
Mycobacterial Infections	1	0.78125	0.375	0.125
	2	0.15625	0.25	0
	3	0.96875	0.125	-0.125
	4	0.625	0.25	0.125
	5	0.53125	0.5	0.375
	6	0.65625	0.125	0.25
	7	0.46875	0.75	0.25
	8	0.40625	0.375	-0.375
	9	0.34375	0.625	0.375
	10	0.78125	0.375	0.375
	11	0.59375	0.25	0.125
	12	0.78125	0.375	0.125
	13	0.8125	0.375	0.25
	14	0.46875	0.375	-0.25
	15	0.65625	0.75	0.25
	16	0.40625	0.25	0
	17	0.5	0.375	0.25
	18	0.53125	0.375	0.25
	19	0.6875	0.125	0.5
	20	0.21875	0.25	0
Fungal Infections	1	0.4375	0.5	0
	2	0.65625	0.375	0
	3	0.4375	0.625	0.125
	4	0.40625	0.25	0.125
	5	0.6875	0.625	-0.125
	6	0.1875	0.125	0.25
	7	0.375	0.375	0.25
	8	0.1875	0.5	-0.125
	9	0.25	0.25	-0.125
	10	0.4375	0.5	0.25
	11	0.21875	0.25	0
	12	0.34375	0.25	-0.25
	13	0.4375	0.25	0.25
	14	0.4375	0.625	0
	15	0.34375	0.375	0.125
	16	0.375	0.625	0.125
	17	0.15625	0.25	-0.25
	18	0.3125	0.25	-0.125
	19	0.5	0.5	0.75
	20	0.5	0.25	0.125
Human Immunodeficiency Virus Infection	1	0.09375	0.25	0.125
	2	0.5	0.625	0
	3	0.40625	0.625	0.125
	4	0.15625	0.25	0
	5	0.15625	0.5	-0.375
	6	0.5	0.5	0
	7	0.25	0.5	0
	8	0.34375	0.5	0
	9	0.3125	0.125	0.25
	10	0.25	0.5	0.375
	11	0.25	0.625	-0.125
	12	0.25	0.5	0.125
	13	0.1875	0.125	0
	14	0.25	0.5	0.25
	15	0.34375	-0.25	-0.125
	16	0.71875	0.5	0.125
	17	0.375	-0.125	0
	18	0.03125	0	0
	19	0.40625	0.625	0.125
	20	0.9375	0.25	0
Viral Arthritides	1	0.375	0.375	0
	2	0.3125	0.375	0.125
	3	0.375	0.875	0
	4	0.15625	0.125	-0.25
	5	0.4375	0.625	0.125
	6	0.34375	0.25	0.375
	7	0.28125	0.375	0
	8	0.25	0.5	-0.5
	9	0.3125	0.375	0
	10	0.53125	0.375	0.375
	11	0.34375	0.375	-0.125
	12	0.3125	0.25	0
	13	0.40625	0.5	0.25
	14	0.4375	0.625	0
	15	0.28125	0.375	-0.125
	16	0.59375	0.625	0
	17	0.28125	0.625	0
	18	0.40625	0.25	-0.125
	19	0.53125	0.625	0.125
	20	0.34375	0	-0.125
Benign Lymphoproliferative & Hematologic Disorders	1	0.21875	0.25	-0.125
	2	0.25	0.125	0.375
	3	0.3125	0.5	0
	4	0.40625	0.5	-0.25
	5	0.21875	0.125	0
	6	0.21875	0.375	0.25
	7	0.3125	0.125	-0.125
	8	0.09375	0.375	0.125
	9	0.5	0.625	0
	10	0.53125	0.5	-0.25
	11	0.375	0.375	0.125
	12	0.40625	0.625	0
	13	0.28125	0.125	0.125
	14	0.375	0.375	0
	15	0.40625	0.625	0.25
	16	0.34375	0.625	0.125
	17	0.53125	0.625	0.375
	18	0.40625	0.5	0.375
	19	0.34375	-0.125	0.375
	20	0.28125	0.625	-0.125
Paraneoplastic & Chemotherapeutic Agents	1	0.3125	0.375	0.125
	2	0.25	0.125	0
	3	0.375	0.375	-0.125
	4	0.5625	0.75	0.375
	5	0.46875	0.25	0.5
	6	0.53125	0.125	0.375
	7	0.46875	0.625	-0.25
	8	0.46875	0.125	0.125
	9	0.40625	0.5	-0.125
	10	0.5	0.5	0
	11	0.3125	0.875	0
	12	0.125	0.25	0.125
	13	0.28125	0.5	-0.5
	14	0.5625	0.75	0.125
	15	0.25	0.375	-0.625
	16	0.4375	0.625	0.25
	17	0.3125	0.625	0
	18	0.28125	0.375	0
	19	0.28125	0.125	-0.25
	20	0.34375	0.375	-0.125
Bony & Cartilaginous Neoplasms	1	0.59375	0.375	0
	2	0.15625	0.25	0.25
	3	0.65625	0.5	0.125
	4	0.40625	0.375	0.5
	5	0.46875	0.5	0.625
	6	0.375	0.375	0.25
	7	0.40625	0.75	0.375
	8	0.4375	0.625	0.5
	9	0.40625	0.375	0.25
	10	0.375	0.125	0.125
	11	0.5	0.625	0.875
	12	0.46875	0.375	0
	13	0.3125	0.625	0
	14	0.4375	0.375	0
	15	0.28125	0.375	0.125
	16	0.46875	0.25	0
	17	0.5	0.375	0
	18	0.4375	0.5	0.75
	19	0.40625	0.375	0.125
	20	0.53125	0.5	0

Finally, test items were grouped by Bloom’s taxonomy in order to calculate their difficulty and discrimination indices. The mean difficulty indices for each group varied from 0.405 (Evaluation) to 0.715 (Comprehension), with significant variability within the six groups. The mean discrimination indices also ranged widely, from 0.220 to 0.548 (Table 5). 

**Table 5:** Test item statistics categorized by Bloom’s Taxonomy.

Taxon	Definition	Skills	Number of Items	Mean Difficulty Index ( Standard Deviation)	Mean Discrimination Index ( Standard Deviation)
Knowledge	Remember facts, terms and basic concepts	Define ; Recognize; Identify	243 (12.78%)	0.677 (0.454)	0.220 (0.223)
Comprehension	Construct meaning from provided representation	Describe; Relay; Recall	571 (30.03%)	0.715 (0.481)	0.244 (0.228)
Apply	Put information into use or practice	Apply; Classify; Illustrate	447 (23.51%)	0.539 (0.488)	0.244 (0.226)
Analysis	Contextualize answers, build connections, and discriminate relevant information	Analyze; Correlate; Distinguish; Compare; Inspect	302 (15.89%)	0.476 (0.497)	0.548 (0.227)
Evaluation	Appraise information to obtain an answer	Evaluate; Appraise; Judge; Consider	239 (12.57%)	0.405 (0.490)	0.268 (0.443)
Synthesis	Generate or construct solutions	Hypothesize; Generate; Design	98 (5.16%)	0.427 (0.492)	0.276 (0.447)

## Discussion

The investigators were able to draft a set of 1900 multiple choice items to evaluate knowledge of rheumatology among trainees. Based on the sampling of these 32 individuals, these items may be suitable for uses like exam preparation review and formative evaluation during training. The systematic manner in which these items were constructed and evaluated has been essential for ensuring transparency of results.

Systematic and methodical test item drafting enables rapid assessment of knowledge 

First, the items were constructed in a rigorous and systematic manner based on established principles of test item creation. The tethering of items to certain principles like simplicity, objectivity, positivity, and clarity helped to prevent the introduction of unwarranted variability associated with test-taking. This allowed for the moderately high-reliability indices that were observed among different sections, as well as the high-reliability index for the entire test-bank.

The approach of defining objectives first, followed by drafting items, was also important in upholding the quality of items. Indeed, it helped to ensure diversity of items, ranging from simple recollection to higher-order evaluation. Additionally, this approach also helped to reduce redundancy that would affect the psychometric properties of test items.

Because the items have relatively short stems and answer choices, these items are particularly useful for rapid assessment of knowledge. These can be used in point-of-care settings where deficits in knowledge can be identified and corrected promptly. Additionally, because each item is explicitly tethered to one or two educational objectives, it enables the test-taker to better recognize the specificity of the defect in knowledge in the context. The explanations and references that accompany each item are also instrumental in ensuring that learners can expand their knowledge base.

The relatively high inter-rater reliability of each criterion speaks to the objectivity to which these criteria were drafted. The criterion with the lowest inter-rater reliability was parallelism, which is understandable given that this is the most difficult criterion to operationalize.

Difficulty and discrimination indices suggest its utility for a variety of learners

Secondly, the items in the item bank tended to be difficult. The mean difficulty index was 0.576. Since one of the principles was setting the difficulty for a second year fellow approaching graduation, a relatively low difficulty index was somewhat expected with a target of 0.65. This has important implications for application. This allows for more opportunities to correct deficits in knowledge. The overall spread and distribution of difficulty indices are also notable, since it allowed the item bank, as a whole, to evaluate test takers of differing abilities. Although the majority of test-takers were rheumatology fellows, this wide variety in difficulty indices may render the item bank suitable for other learners, including medical students, Internal Medicine residents, and physicians in independent practice.

Similarly, the discrimination indices with respect to section scores were modest. Forty-two items (2%) of items had negative discrimination indices with respect to section scores. The distribution of discrimination indices suggests that individual test items can be broadly used to help distinguish low-scorers and high-scorers of sections. As expected, when discrimination indices were calculated with respect to total scores, the values were lower and there were 224 (11.2%) that were negative. This likely reflects the heterogeneity of test-takers who may have strengths in certain sections but weaknesses in others. These items were retained in the bank, but those with negative discrimination indices are marked accordingly in the explanations.

Test items can address multiple levels within Bloom's Taxonomy

Thirdly, there did not appear to be major differences in the difficulty or discrimination indices among the different taxons of objectives. Items focusing on comprehension of rheumatology knowledge were, on average, easier than those focusing on other skills, but the variability was sufficiently high that the mean difficulty indices of all six taxons approximated one another. Similar results were present for the discrimination indices, although it appears that analysis items had, overall, a greater ability to discriminate between high- and low-achievers. This also bolsters the utility of these items to evaluate a host of skills in addition to knowledge and comprehension.

Implications and future directions

This item bank can be utilized in a variety of ways by both learners and teachers. Although most multiple-choice items are more commonly used for summative evaluation purposes (determination of an outcome at the end of an educational program), the sheer number and diversity of items within this item bank may also render this test item bank useful for formative evaluation purposes (assessment of abilities at a given time during the educational program).

Previous studies have suggested that multiple-choice items may be used for self-directed and self-regulated learning [12]. Because each item is linked to a specific learning objective, an explanation, and a reference, these items are particularly suitable for those trainees that would like more structure to their self-directed learning. Additionally, because data is available regarding test-taker performance, self-directed learners can identify their own strengths and weaknesses with regards to their peers. Alternatively, items from this bank may also be used for CME-directed activities.

Beyond the level of the individual, these items may be used in board preparation. Indeed, the item content was loosely based on the American Board of Internal Medicine’s Blueprint for the Certification Examination, with expansions to accommodate further topics relevant to the practice of rheumatology. Since the items are grouped thematically into sections, training programs can use the sections to evaluate trainee performance and adjust didactic and other teaching activities accordingly. These sections have sufficient reliability to help provide a rough estimate on trainee performance with respect to peers.

In addition, future versions of the question bank may include images and videos. They were deliberately excluded in this first version to streamline the process of drafting the test item stems and homogenize test items for psychometric analysis. However, these can be included in future versions with their own guidelines to help ensure sufficiently high difficulty and discrimination indices.

Although these items were distributed electronically as sections and manually graded, there exists the potential for computer-adaptive testing [13]. Computerized testing enables a platform that is friendlier to the test taker and the capacity to instantly grade and report scores. Additionally, sophisticated computer algorithms based on item response theory may be able to utilize the difficulty and discrimination indices to identify the most appropriate items to evaluate trainee performance. Since most of these algorithms select items that the given test-taker has a 50% probability of answering correctly, this is exceptionally suitable for this item bank, since the median and mean difficulty indices for items are 0.563 and 0.576, respectively.

Limitations and unanswered questions

The prospective design of this investigation, the methodological rigor of assessing test item quality, and the relatively large number of participants bolster the validity of this item bank. At the same time, there are notable limitations. 

First, the inclusion of option D ("I will have to look that up") is a deviation from standard test item writing principles. The preferred approach is to recommend that test-takers skip questions altogether. However, since these questions were developed for an audience of learners rather than examiners, the authors felt that it was important to provide an option for learners to reflect on their own degree of self-confidence. While the inclusion of option D likely reduced the reliability of the test, this disadvantage is off-set by benefits for learners who seek to gauge their self-confidence in applying their knowledge. Because we did not force learners to answer all items (instead of 'skipping'), there is ambiguity in interpreting the reasons for a test-taker answering a question as D or skipping. Likewise, for simplicity, we did not split this into other options to assess different levels of metacognition since that was not our intended purpose.

Secondly, our convenience sampling makes it unclear if this data is generalizable to a larger number of rheumatology fellows and other learners. Similarly, our strict guideline that delivery of further test item sections would be contingent on completion of previous sections may have enabled a very high completion rate, but is not likely to be replicable in the general population, where such strictness is unfeasible. Of note, we also did not record performance outcomes in passing board certification or recertification examinations. Therefore, its utility in examination preparation remains unclear.

Thirdly, there are other systemic confounders that may have altered test scores. Because learners were not supervised while taking the items, they may have had access to resources. They were also asked not to guess in favor of answering 'D' (I will have to look that up') or skip the question altogether, but it is likely that many of these answers were products of educated guessing, altering some of our test statistics.

Fourth, though objectives for test items included all six taxons, they were not equally distributed among the taxons. There were fewer test items for analysis, evaluation, and synthesis compared to knowledge, comprehension and application. The reason is likely because the 10 criteria constrained long, nuanced stems that would lend themselves more naturally towards more higher-order thinking. To balance this, the investigators sought to transfer this nuance to the explanations, which go into greater depth for higher-order thinking.

Lastly, rheumatology is an evolving field, and, as it evolves, the item bank will need to be periodically updated to incorporate the most recent state of the art. Likewise, we acknowledge that 42 test items (2%) had negative discrimination indices with respect to section scores. These can be eliminated or significantly modified in future versions. We have continued to include them in the current test item bank, with an advisory about their negative discrimination indices, for full transparency to reviewers and to test-takers.

## Conclusions

A rigorous methodology, employing best practices in test item writing, was used to create the first freely-accessible and reliable test item bank for the assessment of rheumatology knowledge. This test item bank has psychometric properties that may make it suitable for formative evaluation of learners and for self-regulated learning. The test item bank covers the spectrum of rheumatology topics, as set by the American Board of Internal Medicine, and assesses learners across the six taxons of Bloom's taxonomy (knowledge, comprehension, application, analysis, synthesis, and evaluation). Because there has been transparency in the development and the psychometric analysis of these test items, learners using this test item bank are better able to appraise their own performance relative to their peers, and, in so doing, are better empowered to guide their own learning.
